# The Molecular Properties of Peanut Protein: Impact of Temperature, Relative Humidity and Vacuum Packaging during Storage

**DOI:** 10.3390/molecules23102618

**Published:** 2018-10-12

**Authors:** Xiaotong Sun, Hua Jin, Yangyang Li, Haiying Feng, Chunhong Liu, Jing Xu

**Affiliations:** College of Science, Northeast Agricultural University, Harbin 150030, Heilongjiang, China; sunxt0523@163.com (X.S.); jinhua@neau.edu.cn (H.J.); ccwasdrdxnlyy@126.com (Y.L.) 18745724605@163.com (H.F.); liuchunhong@neau.edu.cn (C.L.)

**Keywords:** peanut protein, storage, secondary structure, properties

## Abstract

This study aimed to investigate the variation of molecular functional properties of peanut protein isolate (PPI) over the storage process and reveal the correlation between the PPI secondary structure and properties in the storage procedure. After storage, the molecular properties of PPI changed significantly (*p* < 0.05). Extending storage time resulted in a decrease in free sulfhydryl content, fluorescence intensity, surface hydrophobicity and emulsifying properties, which was accompanied by an increase in protein particle size. The results of infrared spectroscopy suggested the content decline of α-helix and β-sheet, and the content rise of β-turn and random coil. Based on bivariate correlation analysis, it was revealed that surface hydrophobicity and emulsifying activity of PPI was significantly affected by α-helix and by β-turn (*p* < 0.05), respectively. This research supplied more information for the relationship between the peanut protein’s secondary structure and functional properties over the stored process.

## 1. Introduction

Peanut is one of the main oilseed crops in China. In the process of oil extraction, a large amount of defatted peanut flour (DPF) is produced. DPF is rich in protein, which can be used to extract peanut protein isolate (PPI), so as to improve its application value [1]. The nutritional value of peanut protein is high and resembles animal proteins. Moreover, peanut protein contains many essential amino acids that are easily ingested by the human body [2]. Therefore, peanut protein has been widely used as an important replacement of animal source proteins for food production. 

Besides the high nutritional value, the functional properties of PPI also have great influence in its deep processing. These properties include solubility, emulsibility, water-binding capacity, surface hydrophobicity, and rheological properties [3]. The functional properties of protein are largely determined by their structures, while the structural changes are affected by processing and storage conditions [4]. Sheng et al. [5] monitored the structural and functional properties of ovalbumin at 22 °C for 50 days and found that the secondary structure changed from order to disorder. Meanwhile, a more hydrophobic environment for tryptophan was observed, and emulsifying and foaming properties of ovalbumin were reduced due to structural changes during storage. In addition, Wang et al. [6] reported the decreased foaming volumes and the increased foaming stability of gliadin solution during −20 °C storage. Furthermore, Kehrberg and Johnson’s [7] studies had shown that the browning rate of dried sweet cheese whey powder increased with increasing moisture content at room temperature, ranging from 3.6 to 5.5%. Therefore, it can be seen that temperature and relative humidity are important factors affecting the storage stability of protein. On the other hand, the application of vacuum packaging, in which food does not contact with oxygen, is also a common food preservation method. Zakrys-Waliwander et al. [8] showed that high O_2_ concentration induced myosin changes and intermolecular cross-linking in the packaging of beef steaks. Meanwhile, the disulphide bonds were significantly formed and protein oxidation was deeper than that in vacuum-packed samples.

As mentioned above, it can be recognized that molecular functional properties of PPI will also be affected by storage temperature, relative humidity and packaging method. However, accurate information about the relationship between the secondary structure and functional properties of PPI under different storage conditions is still quantitatively limited. Therefore, we studied the effects of different temperature (−20 °C, 4 °C and 37 °C), relative humidity (RH) (33% and 74%) and vacuum packaging (VP) on the molecular properties of PPI. The relationship between secondary structure and functional properties of PPI during storage was also studied by bivariate correlation analysis. These results would provide useful information for peanut protein products from different storage conditions to apply in the food industry.

## 2. Results and Discussion

### 2.1. Structural Characterzation of PPI 

#### 2.1.1. Free Sulfhydryl Content 

**Figure 1:**
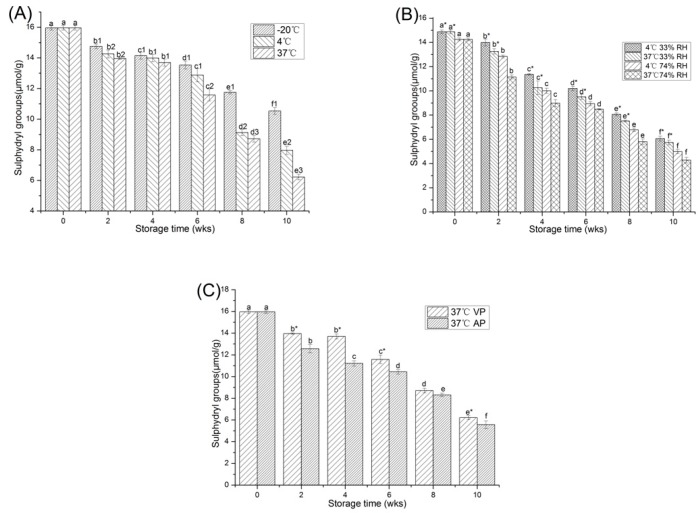
Changes in the free sulfhydryl content of PPI stored at different temperature (**A**), relative humidity (**B**) and packaging methods (**C**). a–f means the significant difference between the same treatments with different weeks (*p* < 0.05). 1–3 means the significant difference between the same weeks with different temperature (**A**) (*p* < 0.05) * means the significant difference between the same weeks and temperature with different relative humidity (**B**) or between the same weeks with different packaging methods (**C**) (*p* < 0.05).

Cysteine residues are the most susceptible amino acid residues of proteins [9], as the change of free sulfhydryl (SH) content in samples is a symbol of cysteine oxidation. Effects of storage time and temperature on free sulfhydryl content of peanut protein were illustrated in Figure 1A. The free sulfhydryl content of peanut protein was initially 15.96 ± 0.15 μmol/g. An evident decrease was observed during 10 weeks of storage in every group (*p* < 0.05) and the highest decline (to 6.22 ± 0.18 μmol/g) occurred in the group of 37 °C. The free sulfhydryl content of different temperature after 10 weeks of storage was in the order as follows: −20 °C (10.54 ± 0.24 μmol/g) > 4 °C (7.97 ± 0.27 μmol/g) > 37 °C (6.22 ± 0.18 μmol/g). During storage, owing to the special sensitivity of free sulfhydryl to oxidation, the sulfur-containing amino acid side chains were converted to disulfides by forming disulfide bonds [10]. Therefore, the number of disulfide bonds increased, while the content of free sulfhydryl groups decreased [11]. The difference in the degree of oxidation among stored samples at different temperatures was due to changes in reaction kinetics [12]. The reaction rate of protein sulphydryl oxidation state depended on storage temperature; therefore, storing PPI at −20 °C delayed the oxidation of PPI. Similar results reported that a reduction in free sulfhydryl of soy protein isolate occurred after storage at 37 °C for 12 weeks, and the decreased free sulfhydryl group was mainly due to the formation of disulfide bonds by oxidation [13]. 

The effect of different water activities on free sulfhydryl content of PPI was shown in Figure 1B. The results showed that free sulfhydryl content of PPI decreased sharply during 10 weeks of storage in high temperature (37 °C) and RH (74%). The free sulfhydryl groups of fresh protein might be relatively stable and less aggregated. However, at high temperature and RH storage, molecular flexibility was minimized, and due to the hydrophobic interactions, the aggregation of unfolded proteins became prominent, enhancing the recombination of S-S bond [14]. Therefore, the content of free sulfhydryl groups decreased rapidly (*p* < 0.05).

The effects of storage time and different packaging methods on free sulfhydryl content of peanut protein were illustrated in Figure 1C. The contents of free sulfhydryl groups stored in VP and AP samples for 10 weeks were significantly reduced (*p* < 0.05). After 10 weeks of storage, the decrease in free sulfhydryl contents in the AP samples (65.1%) was greater than that in the VP samples (61.0%). Protein powder storage studies had shown that even if the protein was sealed in a vacuum or inert gas package, the protein could oxidize as long as the headspace oxygen content reached 1% [10]. Therefore, in the VP group, a small amount of oxygen left resulted in the peanut protein being oxidized. Due to the higher oxygen content present in the AP group, the free sulfhydryl content in AP decreased faster than VP. Lund et al. [15] reported that pork’s meat stored in a package with high concentration of oxygen was consistent with this phenomenon, and other report showed that meat under high concentration of oxygen usually accelerated the decrease in free sulfhydryl content [16]. All data analyzed in this figure are included in the Supplementary Information files.

#### 2.1.2. Secondary Structure Content Determination

During storage, different conditions resulted in significant and complex intermolecular interactions among PPI. These changes affected the conformation of PPI secondary structure including α-helix, β-turn, β-sheet, and random coil as shown in Table 1 and Table 2. Effects of different temperature and packaging methods on the secondary structure of PPI during storage were shown in Table 1. It could be seen that initial peanut protein consisted of 14.7 6± 0.04% α-helix, 36.76 ± 0.07% β-sheet, 34.23 ± 0.07% β-turn, and 14.25 ± 0.04% random coil. The loss of β-sheet was larger than α-helix during storage (Table 1), which might be caused by the significant destroy of intermolecular hydrogen bond in β-sheet [17,18,19]. At the same time, an increased content of β-turn and random coil structure were observed. This finding can be related to proteins oxidation occurring during storage, leading to unfolding, dissociation and rearrangement [20]. Safonova et al. [21] revealed that the oxidation of gluten could lead to elevated contents of β-turn and random coil structure. In brief, based on the data in Table 1, the secondary structure of peanut protein changed from ordered (α-helix and β-sheet) to disordered (β-turn and random coil) conditions. 

The effects of different moisture content and temperature on the secondary structure of PPI during storage were shown in Table 2. After 10 weeks of storage, several structural changes occurred in the PPI. These structural changes were more pronounced at high relative humidity (74% RH) compared to 33% RH. The hydrophilic amino acid residue of the α-helix forms hydrogen bonds with water molecules in the presence of more water, suggesting a series of dimerization or polymerization. The increased moisture content might also lead to intermolecular cross-linking, moving a large number of β-sheet into the interior of molecules [22]. As a result, the contents of α-helix and β-sheet were reduced. At the end of storage (10 weeks), the contents of β-turn and random coils were increased, which indicated that the possible aggregation disrupted the natural monomer and dimer form of protein [23]. These changes in secondary structure were particularly pronounced in samples stored at 37 °C and 74% RH.

#### 2.1.3. Fluorescence Spectroscopy 

**Figure 2:**
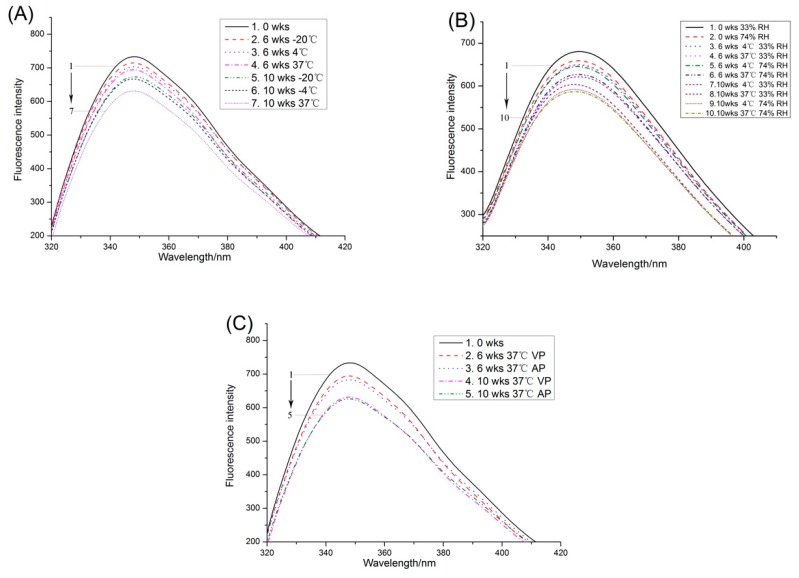
Changes in the intrinsic fluorescence spectra of PPI stored at different temperature (**A**), relative humidity (**B**) and packaging methods (**C**).

Fluorescence spectroscopy is a well-established technique used to characterize changes in the tryptophan (Trp) environment of protein. It is an important indicator of protein conformation and amino acid deletion changes [24]. The change in the tertiary structure of protein can be expressed by the maximum fluorescence emission wavelength of tryptophan, which expresses the relative position of the tryptophan residue in the protein [17]. The fluorescence spectra (Figure 2A) of the storage time and different temperature on protein were obtained to assess conformational transitions around Trp residues. As the storage time progressed, the maximum tryptophan fluorescence intensity gradually decreased, and the maximum emission wavelength occurred at blue shift. After 10 weeks, the intrinsic fluorescence intensity decreased by 8.2% (−20 °C), 9.1% (4 °C) and 13.9% (37 °C). When the microenvironment of tryptophan fluorescence turned less polar, the maximum fluorescence intensity decreased (Figure 2A), which might be caused by peanut protein aggregation during storage. The maximum emission wavelength (λ_max_) of tryptophan fluorescence was susceptibly influenced by its local environment. The blue shifts of the fluorescence emission maximum (λ_max_) during storage suggested that the tryptophan in protein was forced to transfer to the internal non-polar environment. These changes were related to the oxidation of proteins during storage. Wu et al. [9] found the same phenomenon that the oxidation could lead to the formation of soybean protein aggregates, fluorescence intensity of soy protein being decreased, and the blue shift of the maximum emission wavelength being observed.

In Figure 2B, the fluorescence emission maximum of PPI occurring at 349.5 nm to 348 nm was observed, and the decrease of fluorescence intensity was greater at high RH (74%) storage. The results of Katekhong and Charoenrein [25] indicated that in high RH, the egg white protein molecules in the sample were easier to form aggregates during storage, and the proportion of aggregates increased with increasing storage time. The constant aggregation led to the burial of amino acids and a more nonpolar microenvironment of amino acids. Therefore, the fluorescence intensity reduction of high water activity was significantly higher than that of PPI samples with low moisture content.

Intrinsic fluorescence spectroscopy of peanut protein stored in VP and AP was shown in Figure 2C. As the storage time increased, the fluorescence intensity of the samples stored by both packaging methods decreased. During the 10 weeks storage period, the fluorescence intensity of the AP samples decreased by 14.6%, while a smaller decrease of 13.9% was observed in the VP samples. These results suggested that the use of VP inhibited protein oxidation during storage. 

#### 2.1.4. Distribution of Particle Size

**Figure 3:**
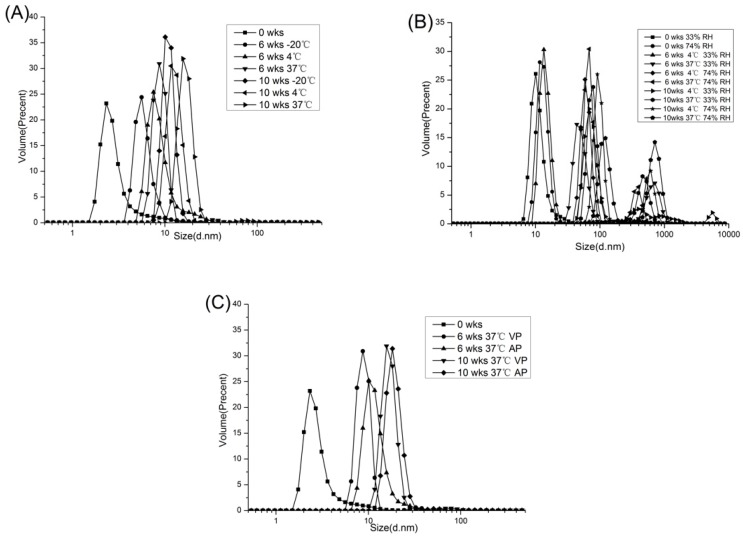
Changes in particle size volume distribution of PPI stored at different temperature (**A**), relative humidity (**B**) and packaging methods (**C**).

Dynamic Light Scattering (DLS) is a sensitive and effective quantification technique used to detect the formation of protein aggregates [26]. Changes in particle size volume distribution of peanut protein with different storage temperature and time were shown in Figure 3A. The results showed that particle size was significantly affected by both storage temperature and time (*p* < 0.05). With the extension of storage time, the particle sizes of peanut protein under three storage temperatures (−20, 4, 37 °C) all became larger, and the growth at 37 °C was more significant. The change of free sulfhydryl content in storage process could explain this phenomenon. From the data in Fig 1, with increasing storage time and temperature, the content of free sulfhydryl groups decreased, indicating an increase in S-S content in the protein. The S-S bonds formed in the protein would cause protein aggregation, thereby increasing the particle size of peanut protein [17]. 

Figure 3B showed the particle size volume distribution for PPI with different water activities during storage. The initial PPI showed a monomodal distribution at 0 weeks of storage (peak at 10.1 nm of 33% RH and 11.7 nm of 74% RH). At high RH (74%), the volume distribution of PPI was bimodal after 10 weeks of storage. And, as RH increased, the distribution shifted to higher sizes. Because higher levels of protein hydration led to higher molecular motility, the high moisture content of protein could accelerate aggregation reactions [27]. Liu et al. [28] observed that the aggregation of protein occurred in freeze-dried bovine serum albumin after storage for 24 h at 37 °C and 96% RH. This moisture-induced aggregation was caused by the formation of intermolecular S-S bridges by thiol-disulfide reaction.

Particle size volume distribution of peanut protein stored in VP and AP was shown in Figure 3C. During the 10 weeks of storage, the particle size distribution of the PPI stored in both packages shifted to the large size direction. However, at the same storage time particle size of VP group was lower than AP group, which suggested that the vacuum packaging delayed the aggregation of protein to some extent. It could be attributed to the finding that the peanut protein stored in the vacuum packaging produced lower reactive oxygen species, and thus the level of protein oxidation was lower.

### 2.2. Molecular Properties of PPI

#### 2.2.1. Surface Hydrophobicity

**Figure 4:**
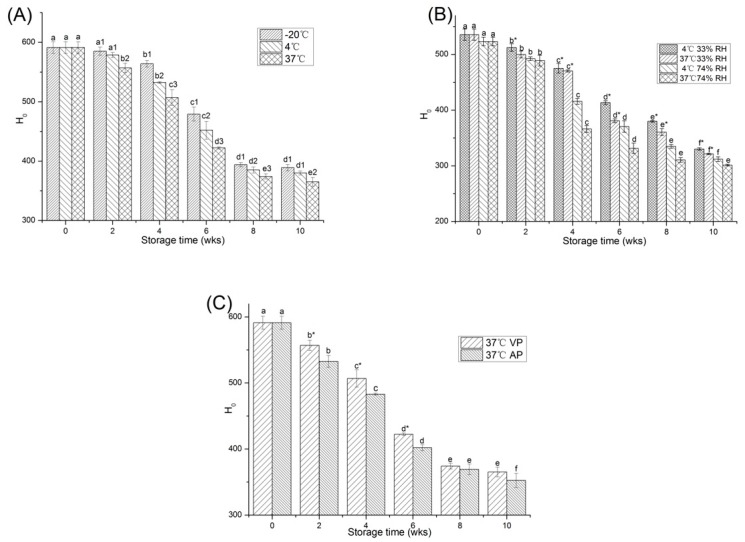
Changes in the H_0_ of PPI stored at different temperature (**A**), relative humidity (**B**) and packaging methods (**C**). a–f means the significant difference between the same treatments with different weeks (*p* < 0.05). 1–3 means the significant difference between the same weeks with different temperature (**A**) (*p* < 0.05). * means the significant difference between the same weeks and temperature with different relative humidity (**B**) or between the same weeks with different packaging methods (**C**) (*p* < 0.05).

The H_0_ of protein reflects the distribution degree of hydrophobic residues of amino acid on the protein surface and is an important molecular characteristic [29]. As can be seen from the Figure 4A, the initial protein H_0_ was 591.38 ± 9.85 for all temperatures. After 10 weeks of storage, the H_0_ of all groups was significantly reduced (*p* < 0.05). Previous studies had shown that the reduction of H_0_ might be due to the oxidation of hydrophobic groups on the surface of proteins [30]. The decrease in H_0_ indicated that protein oxidation occurred during PPI storage. The maximum reduction was found in the high temperature PPI and decreased to 365.24 ± 7.31 during the 10 weeks of storage. Therefore, −20 °C was the most suitable storage temperature, which delayed protein oxidation to the greatest extent. 

In general, the H_0_ decreased as water activity increased (Figure 4B). After 10 weeks of storage, the H_0_ of PPI in each group gradually decreased, and the decrease was more pronounced during the storage of 74% RH at 37 °C (*p* < 0.05). The decrease in H_0_ during storage was caused by the protein aggregation, which was accelerated under high moisture content and high temperature [31].

The effect of different packaging methods on the H_0_ of PPI samples was shown in Figure 4C. The H_0_ of PPI stored by the two packaging methods reduced significantly after 10 weeks (*p* < 0.05). After storage, the reduction in the H_0_ of the AP samples (40.4%) was greater than that of the VP samples (38.2%). This could be attributed to the finding that reactive oxygen species were more likely to cause oxidation of amino acid side chains and backbones of the AP samples, leading to cross-linking among proteins, which affected the H_0_ of protein [11]. In addition, it could be seen that the speed of the H_0_ decrease was not uniform. After 6 weeks, the decrease of the H_0_ values decelerated. This phenomenon may be attributed to the slower oxidation speed, when the PPI was fully oxidized after long-term storage.

#### 2.2.2. Emulsifying Properties

**Figure 5:**
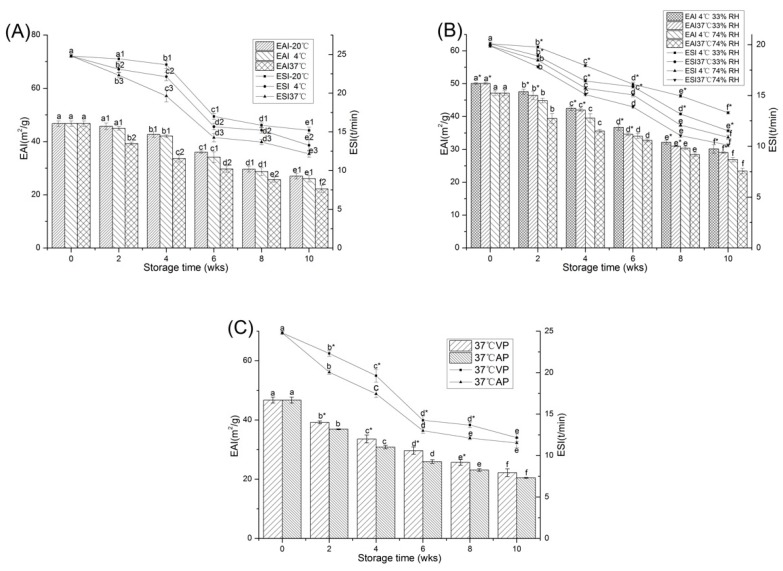
Changes in the EAI and ESI of PPI stored at different temperature (**A**), relative humidity (**B**) and packaging methods (**C**). a–f means the significant difference between the same treatments with different weeks (*p* < 0.05). 1–3 means the significant difference between the same weeks with different temperature (**A**) (*p* < 0.05). * means the significant difference between the same weeks and temperature with different relative humidity (**B**) or between the same weeks with different packaging methods (**C**) (*p* < 0.05).

The emulsifying activity index (EAI) and the emulsification stability index (ESI) of protein reflect the ability of emulsifier to form and stabilize small droplets [32]. EAI represents the ability of proteins to promote the formation of emulsion, and ESI demonstrates the ability of emulsion to resist unstable changes such as coalescence, emulsification, flocculation or sedimentation over a defined period of time [33]. Figure 5A showed that the EAI of PPI was initially 46.74 ± 1.02 m^2^/g, and at all storage temperatures the EAI decreased with the prolonging of storage time. At relatively higher temperature (37 °C), the EAI decreased more significantly (*p* < 0.05) during 10 weeks of storage. The trend of ESI was similar to that of EAI. Emulsifying properties are determined by the ability of protein to adsorb on the surface of the oil droplets and the interaction between the protein molecules. The changes in protein emulsifying properties might be due to different molecular structures and surface charges caused by oxidation during storage, affecting the structural rearrangement of protein at the oil-water interface. Zhang et al. [34] showed that the emulsification properties were positively correlated with the H_0_ of protein, the increase in the H_0_ would promote the adsorption of protein on the oil droplets [35]. In this experiment, with the extension of storage time or increasing temperature, the reduction of hydrophobic groups might hinder molecular assembly of protein molecules through hydrophobic interactions and electrostatic interactions [36]. Consequently, the emulsifying properties of PPI declined. 

The EAI of PPI on the 0 weeks of storage was 50.10 ± 0.27 and 47.15 ± 0.74 m^2^/g with 33% and 74% moisture, respectively (Figure 5B). After 10 weeks of storage at 37 °C, the EAI decrease of 42.00% and 50.31% were observed at 33% and 74% moisture, respectively. The ESI had the same trend as EAI. Our research indicated that long-term storage at higher temperature and moisture content resulted in denaturation or aggregation of protein, causing a decrease in the H_0_ of protein, which could limit the PPI emulsification properties.

The EAI and ESI of peanut protein stored in VP and AP conditions were shown in Figure 5C. After 10 weeks of storage, the EAI and ESI of the VP and AP samples were significantly reduced (*p* < 0.05). It should be noted that the reduction in EAI and ESI was greater in AP groups (by 56.2% and 53.5%) than in VP groups (by 52.4% and 50.9%) after 10 weeks of storage. The H_0_ provided consistent data that supported the trend of emulsifying properties as described above. The results showed that oxygen-free packaging to some extent delayed the decline of emulsifying properties.

### 2.3. Correlation Analysis 

To further verify the relationship between PPI secondary structure and properties during storage, the correlation coefficient among the secondary structure, surface hydrophobicity and emulsifying properties was calculated. The correlation coefficient usually indicates the linearly related degree of two variables. A greater absolute value of the correlation coefficient indicates a stronger correlation [37]. The data in Table 3 showed a significant correlation between the α-helix and the PPI H_0_ (*p* < 0.05). At the same time, the β-turn was also significantly correlated with emulsifying activity (*p* < 0.05). Sheng et al. [5] found that ovalbumin (OVA) emulsion stability was correlated with β-sheet (*p* < 0.01), while α-helix was associated with foam stability (*p* < 0.05) in the stored procedures. The difference in correlation between PPI and OVA might be ascribed to different protein structure and storage conditions. Correlation analysis clearly showed that changes in PPI secondary structure could affect surface hydrophobicity and emulsifying properties as storage time increased. In particular, the transformation of the PPI secondary structure was closely related to the protein function.

## 3. Materials and Methods 

### 3.1. Materials 

Defatted peanut flour produced commercially was achieved from Changshou Co., Ltd. (Qingdao, Shandong, China). Ethylene diaminetetraacetic acid (EDTA), sodium dodecyl sulphate (SDS), 5,5′-dithiobis (2-nitrobenzoic acid) (DTNB), and 1-anilino-naphthalene-8-sulphonate (ANS) were obtained from Sigma Chemical Co. (St. Louis, MO, USA). All other chemicals were of analytical grade.

### 3.2. Storage of PPI 

The extraction of PPI was performed using the method described by Gong et al. [1]. The first set of the freshly prepared PPI powders was stored in vacuum-sealed polyethylene bags. These bags were then stored at −20 °C, 4 °C and 37 °C for 2, 4, 6, 8 and 10 weeks, respectively. The second set of the freshly prepared PPI powders was stored in the atmospheric package (AP) at 37 °C for the same time (2, 4, 6, 8 and 10 weeks). The third set of the freshly prepared PPI powders was separately placed in a desiccator containing saturated MgCl_2_ and NaCl solutions. After 10 days equilibrium at 25 °C, PPI was obtained as the relative humidity stable at 33% and 74%, respectively. These samples were stored at 4 and 37 °C for 2, 4, 6, 8 and 10 weeks, respectively. 

### 3.3. Structural Characterization

#### 3.3.1. Determination of Free Sulfhydryl Content 

A slightly modified method based on that reported by Beveridge et al. [38] was used to determine the free sulfhydryl (SH) content. The PPI solution was diluted by 0.1 M phosphate buffer solution (pH 8.0) with the ratio of 1:2 (*v*/*v*). After adding 67 μL of 0.01 M DTNB, the reaction was carried out for 1 h at 25 °C. The absorbance was measured at 412 nm and the free sulfhydryl content was measured using the extinction coefficient of 13,600 M^−1^cm^−1^.

#### 3.3.2. Fourier Transform Infrared Spectroscopy (FTIR) 

2 mg of each PPI sample was mixed with 200 mg KBr. FTIR scanning was performed at room temperature (25 °C) with dry environment by Bruker Vertex 70 FTIR spectrometer (Bruker Optics, Ettlingen, Germany). The scanning conditions were from 400 to 4000 cm^−1^ with a resolution of 4 cm^−1^, and scanning of each sample occured 64 times.

#### 3.3.3. Fluorescence spectroscopy 

The determination of the fluorescence spectrum was modified according to the method of Wang et al. [39]. The PPI solution was diluted to 0.1 mg/mL with 0.01 M phosphate buffer solution (pH 7.0). The emission spectrum between 300–420 nm was scanned at 280 nm excitation wavelength with the fluorescence spectrometer of F-4500 (Hitachi, Tokyo, Japan) at the slits width of 2.5 nm. 0.01 M phosphate buffer solution (pH 7.0) was used as the blank.

#### 3.3.4. Dynamic Light Scattering (DLS) Measurement 

The mean particle size, size distribution and polydispersity index (PDI) of the samples were detected using a dynamic light scattering instrument at 25 °C (Zetasizer Nano-S90, Malvern Instruments, Malvern, UK). Before the detection, all the samples were freshly prepared by adjusting protein concentration to 0.2 mg/mL with 0.01 M phosphate buffer (pH 7.0) to avoid multiple scattering effects.

#### 3.3.5. Determination of Surface Hydrophobicity (H_0_) 

H_0_ was determined by a modified method of Huang et al. [40]. 0.01 M phosphate buffer (pH 7.0) was used to dissolve peanut protein. The solution was magnetically stirred for 1 hour and then centrifuged at 10,000 rpm for 10 min. After that, the up-layer solution was collected and its concentration was detected with the Biuret method [41]. The concentration of protein was then diluted to 0.05–0.5 mg/mL with the same phosphate buffer. To 4 mL of different concentration samples 40 μL of 8 mM ANS were added. The detection of fluorescence intensity was carried out at excitation wavelength 390 nm and emission wavelength 470 nm with an F-4500 fluorometer (Hitachi, Tokyo, Japan). The fluorescence intensity was plotted against the protein concentration, and the slope of the curve was the H_0_ of peanut protein.

#### 3.3.6. Determination of Emulsifying Properties 

A slightly modified method of Pearce and Kinsella [42] was used to determine the emulsifying properties. The peanut protein concentration was adjusted with phosphate buffer (0.01 M, pH 7.0) to 10 mg/mL. 15 mL of protein solution and 5 mL of soybean oil were mixed together. The samples were homogenized at 10,000 rpm for 2 minutes. 20 μL of peanut protein-soybean oil emulsion was uniformly mixed with 5 mL 0.1% SDS. The absorbance (A_0_) was detected at 500 nm with 0.1% SDS as a blank. The emulsion absorbance (A_10_) was measured by the same method after standing for 10 min. Peanut protein emulsifying activity index (EAI) and emulsion stability index (ESI) were calculated as follows:

(1) $$\mathrm{EAI}(m^{2}/g)=\frac{2\times 2.303\times A_{0}\times 250}{c\times 1\times (1-\varphi )\times 10000}$$

(2) $$\mathrm{ESI}\;\left(\min\right)=\frac{A_{0}}{A_{0}-A_{10}}\times t$$

where c was the protein concentration in the sample (10 mg/mL); *ϕ* was the fraction of the oil phase (0.25).

### 3.4. Statistical Analysis 

Experiments were carried out in triplicate. Results were expressed as mean ± standard deviation. Significance of difference between the means was identified through the Duncan′s multiple-range tests (*p* < 0.05) with SPSS 20.0 software (IBM, Chicago, IL, USA). The coefficients reflecting the relationship among secondary structure, emulsifying properties and surface hydrophobicity were determined by Pearson’s correlation analysis.

## 4. Conclusions

The changes in free sulfhydryl content, secondary and tertiary structure of protein indicated denaturation and aggregation during storage. Furthermore, the decrease in PPI surface hydrophobicity and emulsifying properties was also observed, as well as the particle size increase during storage. The results of correlation analysis showed that the α-helix significantly affected the surface hydrophobicity of PPI (*p* < 0.05), while the β-turn significantly affected the emulsifying activity of PPI (*p* < 0.05). These finding suggested that storage would affect the PPI molecules and further affect its functional properties. The results of this study could provide a new insight for the relationship between the peanut protein secondary structure and function properties in storage. In addition, our research would be useful for the function properties utilization of peanut protein products during storage.

## Figures and Tables

**Table 1:** Secondary structure compositions of PPI under different temperatures and packaging methods.

Temperature (°C)	Time (Weeks)		
	0	6	10
**α-Helix (%)**			
−20 °C	14.76 ± 0.04 ^a^	^x^ 14.58 ± 0.07 ^b^	^x^ 14.35 ± 0.05 ^c^
4 °C	14.76 ± 0.04 ^a^	^xy^ 14.43 ± 0.09 ^b^	^x^ 14.31 ± 0.07 ^b^
37 °C	14.76 ± 0.04 ^a^	^y^ 14.39 ± 0.07 ^b^	^x^ 14.29 ± 0.03 ^c^
37 °C AP	14.76 ± 0.04 ^a^	* 14.24 ± 0.05 ^b^	* 14.18 ± 0.07 ^b^
**β-Sheet (%)**			
−20 °C	36.76 ± 0.07 ^a^	^x^ 36.41 ± 0.08 ^b^	^x^ 35.60 ± 0.08 ^c^
4 °C	36.76 ± 0.07 ^a^	^y^ 36.11 ± 0.05 ^b^	^x^ 35.51 ± 0.06 ^c^
37 °C	36.76 ± 0.07 ^a^	^z^ 35.95 ± 0.04 ^b^	^y^ 35.31 ± 0.04 ^c^
37 °C AP	36.76 ± 0.07 ^a^	* 35.62 ± 0.04 ^b^	* 35.17 ± 0.03 ^c^
**β-Turn (%)**			
−20 °C	34.23 ± 0.07 ^c^	^z^ 34.70 ± 0.10 ^b^	^y^ 35.37 ± 0.08 ^a^
4 °C	34.23 ± 0.07 ^c^	^y^ 35.01 ± 0.09 ^b^	^xy^ 35.41 ± 0.10 ^a^
37 °C	34.23 ± 0.07 ^c^	^x^ 35.16 ± 0.05 ^b^	^x^ 35.58 ± 0.08 ^a^
37 °C AP	34.23 ± 0.07 ^c^	* 35.43 ± 0.04 ^b^	* 35.80 ± 0.07 ^a^
**Random Coil (%)**			
−20 °C	14.25 ± 0.04 ^b^	^y^ 14.31 ± 0.03 ^b^	^y^ 14.68 ± 0.05 ^a^
4 °C	14.25 ± 0.04 ^c^	^x^ 14.45 ± 0.06 ^b^	^x^ 14.77 ± 0.03 ^a^
37 °C	14.25 ± 0.04 ^c^	^x^ 14.50 ± 0.03 ^b^	^x^ 14.82 ± 0.02 ^a^
37 °C AP	14.25 ± 0.04 ^c^	* 14.71 ± 0.05 ^b^	14.85 ± 0.04 ^a^

a–c means the significant difference between the same treatments with different weeks (*p <* 0.05). x–z means the significant difference between the same weeks with different temperature (*p <* 0.05). * means the significant difference between the same weeks with different packaging methods.

**Table 2:** Secondary structure compositions of PPI under different relative humidity.

Temperature (°C)	Time (Weeks)		
	0	6	10
**α-Helix (%)**			
4 °C 33% RH	14.69 ± 0.08 ^a^	* 14.48 ± 0.07 ^b^	* 14.33 ± 0.05 ^b^
37 °C 33% RH	14.69 ± 0.08 ^a^	14.35 ± 0.08 ^b^	* 14.27 ± 0.08 ^b^
4 °C 74% RH	14.59 ± 0.10 ^a^	14.30 ± 0.04 ^b^	14.16 ± 0.07 ^b^
37 °C 74% RH	14.59 ± 0.10 ^a^	14.19 ± 0.10 ^b^	14.10 ± 0.03 ^b^
**β-Sheet (%)**			
4 °C 33% RH	36.60 ± 0.09 ^a^	*36.03 ± 0.08 ^b^	* 35.35 ± 0.04 ^c^
37 °C 33% RH	36.60 ± 0.09 ^a^	*35.74 ± 0.04 ^b^	* 35.19 ± 0.05 ^c^
4 °C 74% RH	36.51 ± 0.08 ^a^	35.47 ± 0.08 ^b^	34.96 ± 0.05 ^c^
37 °C 74% RH	36.51 ± 0.08 ^a^	35.26 ± 0.09 ^b^	34.47 ± 0.08 ^c^
**β-Turn (%)**			
4 °C 33% RH	* 34.41 ± 0.09 ^a^	* 35.13 ± 0.10 ^b^	* 35.83 ± 0.06 ^c^
37 °C 33% RH	* 34.41 ± 0.09 ^a^	* 35.50 ± 0.06 ^b^	* 36.02 ± 0.05 ^c^
4 °C 74% RH	34.55 ± 0.08 ^a^	35.74 ± 0.07 ^b^	36.33 ± 0.09 ^c^
37 °C 74% RH	34.55 ± 0.08 ^a^	35.98 ± 0.08 ^b^	36.74 ± 0.07 ^c^
**Random Coil (%)**			
4 °C 33% RH	14.30 ± 0.08 ^a^	* 14.36 ± 0.07 ^ab^	14.49 ± 0.04 ^b^
37 °C 33% RH	14.30 ± 0.08 ^a^	* 14.41 ± 0.06 ^ab^	* 14.52 ± 0.07 ^b^
4 °C 74% RH	14.35 ± 0.10 ^a^	14.49 ± 0.06 ^ab^	14.55 ± 0.04 ^b^
37 °C 74% RH	14.35 ± 0.10 ^a^	14.57 ± 0.10 ^b^	14.69 ± 0.07 ^b^

a–c means the significant difference between the same treatments with different weeks (*p <* 0.05). * means the significant difference between the same weeks and temperature with different relative humidity (*p <* 0.05).

**Table 3:** Correlation analysis between secondary structure and other factors.

	α-Helix	β-Sheet	β-Turn	Random	EAI	ESI	H_0_
**α-Helix**	1						
**β-Sheet**	0.986	1					
**β-Turn**	−0.995 *	−0.997 *	1				
**Random**	−0.968	−0.994 *	0.983	1			
**EAI**	0.993	0.989	−0.996 *	−0.969	1		
**ESI**	0.974	0.969	−0.980	−0.945	0.985	1	
**H_0_**	0.997 *	0.986	−0.994	−0.968	0.994	0.979	1

* indicates significant correlations at *p* < 0.05.

## Supplementary Materials

The supplementary materials are available online.
