# Supplementary material for: The Molecular Properties of Peanut Protein: Impact of Temperature, Relative Humidity and Vacuum Packaging during Storage
Source: Molecules. 2018 Oct 12;23(10):2618. doi: 10.3390/molecules23102618 (PMC6222477; doi:10.3390/molecules23102618)
Supplement: Supplementary file 1 [file molecules-23-02618-s001.zip › supplementary-final/Experimental data.pdf]

Table.1. Changes in PPI free SH

	-20 °C			4 °C			37 °C			37 °C AP		
**0 wks**	16.12	15.83	15.94	16.12	15.83	15.94	16.12	15.83	15.94	16.12	15.83	15.94
**2 wks**	14.91	14.61	14.75	14.54	14.03	14.21	14.03	13.94	13.91	12.97	12.22	12.52
**4 wks**	14.36	13.9	14.19	14.26	13.77	13.94	13.94	13.49	13.66	11.48	10.99	11.16
**6 wks**	13.76	13.26	13.59	13.33	12.46	12.83	11.99	11.22	11.53	10.67	10.26	10.39
**8 wks**	11.88	11.66	11.74	9.35	8.95	9.08	8.94	8.53	8.68	8.48	8.20	8.27
**10 wks**	10.77	10.33	10.51	8.26	7.72	7.92	6.42	6.07	6.17	5.94	5.25	5.52

	4 °C 33%RH			37 °C 33%RH			4 °C 74%RH			37 °C 74%RH		
**0 wks**	15.04	14.75	14.82	15.04	14.75	14.82	14.38	14.17	14.2	14.38	14.17	14.20
**2 wks**	14.30	13.73	13.94	13.49	13.08	13.21	12.97	12.76	12.79	11.33	11.00	11.09
**4 wks**	11.43	11.34	11.31	10.84	9.73	10.21	10.25	9.82	9.96	9.30	8.73	8.94
**6 wks**	10.39	10.04	10.14	9.74	9.33	9.46	9.14	8.8	8.89	8.52	8.51	8.44
**8 wks**	8.20	7.97	8.01	7.59	7.48	7.46	6.96	6.67	6.74	6.05	5.60	5.75
**10 wks**	6.29	5.88	6.01	5.95	5.58	5.69	5.18	4.85	4.94	4.53	4.08	4.23

Table.2. Changes in PPI fluorescence intensity

	0 wks	6wks -20℃	6wks 4℃	6wks 37℃	6wks 37℃ AP	10wks -20℃	10wks 4℃	10wks 37℃	10wks 37℃ AP
**300**	750.46428	540.10074	576.07591	669.94795	450.91739	664.54595	581.03197	619.87156	657.55421
**300.5**	719.57177	519.11099	553.43942	642.72994	433.91181	637.79142	557.99224	598.81152	631.21392
**301**	689.60068	498.81093	531.51928	616.3372	417.494	611.83165	535.66905	578.30502	605.64425
**301.5**	660.551	479.20055	510.3155	590.76974	401.66396	586.66665	514.06238	558.35205	580.84521
**302**	632.42273	460.27987	489.82807	566.02756	386.42169	562.2964	493.17225	538.95263	556.81678
**302.5**	605.21586	442.04887	470.057	542.11066	371.7672	538.72092	472.99865	520.10674	533.55898
**303**	578.93041	424.50757	451.00228	519.01904	357.70047	515.94021	453.54158	501.81438	511.0718
**303.5**	553.56637	407.65595	432.66392	496.75269	344.22152	493.95425	434.80104	484.07557	489.35525
**304**	529.12374	391.49402	415.04192	475.31163	331.33033	472.76306	416.77703	466.89029	468.40931
**304.5**	505.60252	376.02178	398.13626	454.69584	319.02692	452.36663	399.46955	450.25854	448.234
**305**	483.00271	361.23923	381.94697	434.90533	307.31128	432.76496	382.87861	434.18034	428.82931
**305.5**	461.32431	347.14637	366.47403	415.9401	296.18341	413.95806	367.00419	418.65567	410.19524

**306**	440.56732	333.7432	351.71744	397.80014	285.64332	395.94592	351.8463	403.68454	392.33179
**306.5**	420.73174	321.02971	337.67721	380.48547	275.69099	378.72854	337.40495	389.26694	375.23897
**307**	401.81757	309.00592	324.35333	363.99607	266.32644	362.30592	323.68013	375.40288	358.91677
**307.5**	383.82482	297.67182	311.74581	348.33196	257.54965	346.67807	310.67183	362.09236	343.36519
**308**	366.75347	287.0274	299.85465	333.49312	249.36064	331.84497	298.38007	349.33537	328.58423
**308.5**	350.60353	277.07267	288.67984	319.47955	241.7594	317.80665	286.80484	337.13193	314.5739
**309**	335.375	267.80764	278.22138	306.29127	234.74593	304.56308	275.94614	325.48201	301.33418
**309.5**	321.06789	259.23229	268.47928	293.92827	228.32023	292.11428	265.80397	314.38564	288.86509
**310**	307.68218	251.34663	259.45353	282.39054	222.48231	280.46023	256.37833	303.8428	277.16663
**310.5**	295.21789	244.15066	251.14414	271.67809	217.23215	269.60096	247.66922	293.8535	266.23878
**311**	283.675	237.64438	243.55111	261.79092	212.56977	259.53644	239.67665	284.41773	256.08156
**311.5**	273.05353	231.82779	236.67443	252.72903	208.49515	250.26669	232.4006	275.53551	246.69495
**312**	263.35346	226.70089	230.5141	244.49242	205.00831	241.7917	225.84109	267.20682	238.07897
**312.5**	254.57481	222.26368	225.07013	237.08108	202.10924	234.11147	219.9981	259.43166	230.23362
**313**	228.57587	205.03431	205.97484	214.15245	188.30237	210.87355	200.38292	236.26467	206.91339
**313.5**	205.38991	189.766	188.94176	193.70164	176.06875	190.02642	182.85614	212.20668	185.91898
**314**	185.34632	176.72916	174.2631	176.06133	165.66363	171.89714	167.71142	190.56239	167.57687
**314.5**	168.74253	166.15415	162.19091	161.51745	157.30701	156.76902	155.21686	171.66204	152.17923
**315**	155.85026	158.23913	152.947	150.31251	151.19496	144.8936	145.60894	155.80357	139.98563
**315.5**	146.88594	153.154	146.72769	142.63158	147.49643	136.48857	139.07207	143.25374	131.20564
**316**	142.19331	151.24482	143.92609	138.85312	146.56581	131.97415	135.97279	134.22714	126.23353
**316.5**	141.85651	152.5806	144.63255	139.06265	148.44075	131.44875	136.3789	129.1298	125.2007
**317**	145.75779	157.07798	148.75063	143.11073	153.05673	134.7643	140.17504	128.06949	128.00526
**317.5**	153.5965	164.56801	156.06547	150.67565	160.28606	141.61039	147.12946	130.91794	134.35911
**318**	164.77431	174.71699	166.14037	161.17788	169.8627	151.45963	156.83243	137.38068	143.71523
**318.5**	178.57271	187.10121	178.45865	174.02101	181.39704	163.73877	168.81364	146.89794	155.49653
**319**	194.15052	201.13669	192.37245	188.50518	194.39379	177.7223	182.44948	158.8764	168.99763
**319.5**	210.79152	216.24815	207.29642	204.01059	208.36799	192.73697	197.1301	172.59135	183.57348
**320**	227.86009	231.88433	222.68211	219.91313	222.88135	208.1478	212.25834	187.38856	198.57492
**320.5**	244.92156	247.6482	238.16335	235.7924	237.58754	223.5442	227.41761	202.6119	213.56319

**321**	261.67049	263.26031	253.49783	251.38561	252.2302	238.67726	242.33505	217.82545	228.25403
**321.5**	277.99213	278.57885	268.58502	266.62799	266.64523	253.45451	256.88754	232.74328	242.55768
**322**	293.83876	293.53184	283.35828	281.48008	280.75243	267.82212	271.01276	247.26777	256.41233
**322.5**	309.21638	308.11396	297.78485	295.93417	294.53568	281.76858	284.71367	261.32278	269.80162
**323**	324.11297	322.33417	311.82322	309.97718	308.00766	295.29952	298.03321	274.89509	282.71594
**323.5**	338.57758	336.23178	325.48706	323.68111	321.19485	308.47789	311.05677	287.98235	295.24047
**324**	352.66612	349.8264	338.79389	337.09465	334.12818	321.35588	323.828	300.67145	307.43084
**324.5**	366.47796	363.17022	351.80569	350.27231	346.85514	333.99402	336.37256	313.01031	319.34519
**325**	380.06832	376.30384	364.57125	363.20394	359.42191	346.41752	348.68849	325.0485	330.9822
**325.5**	393.50958	389.28291	377.17838	375.93201	371.86145	358.68414	360.81626	336.78655	342.40577
**326**	406.82839	402.12609	389.69456	388.49834	384.18597	370.83764	372.79011	348.2982	353.6691
**326.5**	420.04861	414.85444	402.17224	400.95881	396.40079	382.92362	384.65031	359.65235	364.83711
**327**	433.15442	427.46529	414.60575	413.31454	408.50827	394.93972	396.39786	370.90817	375.91269
**327.5**	446.12667	439.94749	426.95381	425.53732	420.49466	406.86553	408.01825	382.0648	386.87881
**328**	458.9386	452.27607	439.16611	437.60044	432.33284	418.6726	419.49312	393.10125	397.71155
**328.5**	471.5589	464.42339	451.19733	449.48476	443.98713	430.32887	430.8074	403.99926	408.39268
**329**	483.95511	476.36636	463.01446	461.17098	455.43071	441.80741	441.94963	414.74026	418.90486
**329.5**	496.09322	488.09749	474.59184	472.62344	466.64674	453.08057	452.90027	425.31349	429.22037
**330**	507.9382	499.60539	485.91367	483.80807	477.61885	464.1186	463.63559	435.69877	439.30451
**330.5**	519.46422	510.8651	496.96936	494.70908	488.32501	474.88814	474.13074	445.8742	449.12921
**331**	530.66151	521.83734	507.75579	505.32827	498.74717	485.36904	484.37313	455.80939	458.67994
**331.5**	541.52802	532.49413	518.2653	515.67077	508.88067	495.55688	494.35175	465.48617	467.95084
**332**	552.07151	542.82465	528.48927	525.73469	518.7317	505.45744	504.06095	474.88894	476.93652
**332.5**	562.30467	552.832	538.41711	535.51137	528.30956	515.06522	513.47789	484.01212	485.6395
**333**	572.25215	562.52317	548.04522	545.0057	537.6166	524.37232	522.58438	492.83855	494.07106
**333.5**	581.93645	571.90902	557.37271	554.21892	546.64239	533.37247	531.37503	501.36299	502.23759
**334**	591.37501	581.00267	566.40735	563.13939	555.37205	542.07782	539.87703	509.58855	510.13532
**334.5**	600.56312	589.81592	575.165	571.74438	563.79705	550.49949	548.11531	517.53807	517.75411
**335**	609.48485	598.36217	583.6737	580.04169	571.91799	558.64998	556.10722	525.22793	525.10011
**335.5**	618.1163	606.6497	591.95135	588.05225	579.73584	566.5323	563.85117	532.67209	532.18914

**336**	626.45376	614.68186	599.99975	595.79459	587.2507	574.1619	571.35736	539.87622	539.04865
**336.5**	634.50354	622.45394	607.80683	603.27388	594.47371	581.55144	578.63846	546.85918	545.69607
**337**	642.28372	629.95883	615.3699	610.52329	601.43552	588.71122	585.71156	553.64092	552.14014
**337.5**	649.79833	637.18445	622.68379	617.57509	608.17561	595.62965	592.56316	560.23342	558.37415
**338**	657.04681	644.12264	629.73399	624.43216	614.7181	602.29377	599.16933	566.61619	564.38558
**338.5**	664.02027	650.76326	636.4964	631.0478	621.06192	608.69356	605.50331	572.7583	570.15702
**339**	670.73147	657.09909	642.9624	637.37948	627.19187	614.8421	611.56533	578.63128	575.67507
**339.5**	677.18664	663.11942	649.13826	643.39955	633.09393	620.75317	617.35215	584.22497	580.93854
**340**	683.38101	668.82944	655.03811	649.10365	638.75572	626.43115	622.86016	589.52887	585.95301
**340.5**	689.27834	674.24336	660.66653	654.47467	644.15928	631.85685	628.07611	594.53436	590.71935
**341**	694.84555	679.37357	666.01738	659.50226	649.27611	637.01156	632.99956	599.23504	595.23196
**341.5**	700.05709	684.21385	671.07271	664.1908	654.07275	641.88156	637.63651	603.63374	599.47855
**342**	704.89284	688.74897	675.80886	668.57291	658.52178	646.45364	641.99815	607.73814	603.44525
**342.5**	709.32822	692.96302	680.19658	672.66312	662.6043	650.70198	646.07766	611.54571	607.11432
**343**	713.35496	696.84056	684.2123	676.44213	666.30634	654.59707	649.84919	615.04011	610.47577
**343.5**	716.99257	700.36943	687.849	679.87377	669.61763	658.11898	653.27791	618.20687	613.53096
**344**	720.27754	703.53837	691.10924	682.94494	672.52989	661.25876	656.33997	621.0426	616.28187
**344.5**	723.22865	706.33625	693.98611	685.65346	675.04555	664.01695	659.01636	623.53906	618.72136
**345**	725.83744	708.74922	696.46891	687.99693	677.17975	666.38792	661.28897	625.67625	620.82557
**345.5**	728.07469	710.7667	698.56122	689.97029	678.95802	668.36614	663.14212	627.43946	622.57678
**346**	729.90793	712.38022	700.27553	691.57579	680.39765	669.9544	664.58134	628.83648	623.96327
**346.5**	731.31861	713.58768	701.61347	692.81121	681.49726	671.17375	665.6328	629.88845	624.98753
**347**	732.31522	714.39069	702.56594	693.66754	682.25097	672.05239	666.33127	630.61636	625.65394
**347.5**	732.9343	714.80003	703.13175	694.15085	682.66372	672.62037	666.70089	631.03103	625.98381
**348**	733.21545	714.824	703.32816	694.2764	682.74713	672.90651	666.75769	631.146	626.00658
**348.5**	733.18084	714.48335	703.18186	694.05834	682.50702	672.9289	666.5139	630.9772	625.74936
**349**	732.8238	713.80162	702.71369	693.4964	681.95082	672.69094	665.98004	630.53597	625.21966
**349.5**	732.13305	712.81	701.93695	692.59295	681.09027	672.18592	665.15457	629.81832	624.41884
**350**	731.10206	711.51606	700.86136	691.35657	679.93991	671.40522	664.0292	628.81389	623.35449
**350.5**	729.73521	709.9305	699.50039	689.81506	678.51305	670.34068	662.60605	627.52541	622.0479

**351**	728.03064	708.06642	697.85906	687.99534	676.83144	668.98982	660.91283	625.96757	620.5132
**351.5**	725.99679	705.96081	695.93428	685.91382	674.92215	667.35783	658.98832	624.16227	618.7597
**352**	723.65957	703.63876	693.72432	683.57526	672.80431	665.46287	656.86299	622.12546	616.79193
**352.5**	721.06148	701.12211	691.24655	681.00939	670.48	663.32632	654.55164	619.88123	614.63132
**353**	718.23661	698.42294	688.53925	678.27041	667.94609	660.97734	652.07109	617.4527	612.30752
**353.5**	715.21083	695.5715	685.65034	675.41084	665.21039	658.44258	649.44631	614.87198	609.84888
**354**	712.00825	692.59112	682.62967	672.44882	662.30047	655.75311	646.6992	612.163	607.27019
**354.5**	708.66395	689.49918	679.51745	669.3961	659.24439	652.92832	643.83569	609.34848	604.58328
**355**	705.21509	686.28634	676.34518	666.27415	656.0677	649.99035	640.85995	606.43463	601.8085
**355.5**	701.70146	682.95494	673.13326	663.11324	652.79818	646.95955	637.79153	603.42917	598.97147
**356**	698.15715	679.52083	669.89118	659.91695	649.47788	643.86568	634.66291	600.33168	596.09065
**356.5**	694.60088	676.02502	666.6159	656.67013	646.14583	640.72537	631.49504	597.15716	593.17505
**357**	691.03878	672.4971	663.30525	653.35944	642.8199	637.54123	628.29083	593.91848	590.22919
**357.5**	687.46506	668.96467	659.96355	649.99753	639.49522	634.31055	625.04777	590.6383	587.25801
**358**	683.86997	665.44446	656.60596	646.59946	636.15728	631.04967	621.78112	587.33245	584.2604
**358.5**	680.2377	661.94887	653.25326	643.15316	632.79354	627.78464	618.51321	584.02332	581.2369
**359**	676.55802	658.4653	649.92063	639.63489	629.3982	624.53191	615.25716	580.71346	578.19124
**359.5**	672.82744	654.97289	646.60915	636.04896	625.97244	621.2783	612.00653	577.40426	575.13599
**360**	669.05775	651.45457	643.30244	632.42411	622.51697	618.00601	608.75318	574.09519	572.0828
**360.5**	665.26735	647.90442	639.97793	628.7731	619.03088	614.70557	605.49144	570.79186	569.03621
**361**	661.48426	644.31649	636.61671	625.09211	615.5209	611.38745	602.21508	567.48266	565.98717
**361.5**	657.72319	640.68765	633.2137	621.38952	612.00625	608.04493	598.90587	564.15092	562.91801
**362**	653.98514	637.01729	629.76873	617.69049	608.50499	604.65744	595.54508	560.78188	559.81166
**362.5**	650.24922	633.3064	626.28061	613.98535	605.01885	601.20892	592.11494	557.37496	556.65528
**363**	646.49224	629.55312	622.75258	610.22421	601.53431	597.7128	588.61047	553.9305	553.4338
**363.5**	642.68773	625.74668	619.197	606.35495	598.03446	594.18865	585.0354	550.44622	550.14056
**364**	638.81719	621.87285	615.62175	602.37315	594.49574	590.63795	581.40741	546.91618	546.78059
**364.5**	634.84689	617.9214	612.00703	598.30022	590.88957	587.03703	577.73794	543.33945	543.37165
**365**	630.74543	613.89641	608.31666	594.15352	587.18207	583.35929	574.0258	539.72335	539.91725
**365.5**	626.48553	609.79783	604.51867	589.92147	583.34847	579.5921	570.25417	536.07318	536.41024

**366**	622.06856	605.62106	600.60254	585.59691	579.36857	575.73383	566.41065	532.37973	532.83732
**366.5**	617.50814	601.35506	596.56374	581.18599	575.22765	571.77024	562.48809	528.62249	529.19711
**367**	612.83049	596.99043	592.39372	576.69357	570.92041	567.67516	558.47572	524.78753	525.49206
**367.5**	608.05847	592.5083	588.08613	572.08429	566.46816	563.43221	554.34756	520.86642	521.71879
**368**	603.21179	587.89174	583.64966	567.31235	561.91251	559.055	550.07808	516.85673	517.86278
**368.5**	598.28847	583.13076	579.09893	562.36605	557.28645	554.57209	545.66181	512.75809	513.90323
**369**	593.26875	578.22465	574.43699	557.28145	552.5938	549.99978	541.10579	508.57236	509.82407
**369.5**	588.1208	573.17168	569.64863	552.09661	547.82364	545.33751	536.4086	504.29593	505.61701
**370**	582.82562	567.97506	564.728	546.82846	542.96615	540.57673	531.56485	499.92795	501.28528
**370.5**	577.38498	562.64332	559.68048	541.46807	538.00811	535.71737	526.58368	495.47314	496.84202
**371**	571.82301	557.19337	554.51875	536.01	532.92624	530.76602	521.49617	490.94109	492.30803
**371.5**	566.17362	551.6426	549.24663	530.46209	527.71255	525.73213	516.33279	486.33319	487.70802
**372**	560.47253	546.00579	543.86933	524.84371	522.39705	520.6237	511.11374	481.64562	483.06223
**372.5**	554.73459	540.29227	538.39596	519.16244	517.0313	515.44979	505.8537	476.8776	478.382
**373**	548.94878	534.51171	532.84427	513.41018	511.64898	510.22158	500.57181	472.04322	473.67191
**373.5**	543.09449	528.68736	527.22974	507.58775	506.25774	504.94485	495.28572	467.15985	468.92857
**374**	537.1646	522.84685	521.56433	501.71945	500.85852	499.62228	489.99647	462.23508	464.15143
**374.5**	531.17004	517.01617	515.86307	495.8427	495.45554	494.2587	484.68684	457.26426	459.34564
**375**	525.11993	511.20526	510.14661	489.98017	490.04867	488.85974	479.34194	452.24588	454.53048
**375.5**	519.02709	505.41951	504.43459	484.14012	484.63242	483.43572	473.96945	447.19789	449.72099
**376**	512.9147	499.65507	498.73806	478.32439	479.21299	478.0059	468.59015	442.15456	444.92596
**376.5**	506.82421	493.91287	493.06563	472.54692	473.8093	472.59942	463.22181	437.1462	440.15224
**377**	500.79107	488.18912	487.42775	466.8288	468.43957	467.24585	457.88049	432.1842	435.41789
**377.5**	494.83894	482.49105	481.83885	461.19145	463.11196	461.96104	452.59142	427.272	430.74488
**378**	488.9695	476.82748	476.31675	455.63998	457.83594	456.75307	447.38637	422.41809	426.14764
**378.5**	483.19207	471.21983	470.87739	450.17538	452.63025	451.62288	442.2867	417.63275	421.63094
**379**	477.52196	465.68333	465.53097	444.79819	447.51693	446.57759	437.29183	412.9115	417.19453
**379.5**	471.97531	460.23632	460.28432	439.51817	442.50603	441.61655	432.38497	408.24142	412.83751
**380**	466.54549	454.88544	455.14001	434.33413	437.59711	436.74189	427.55559	403.61303	408.55719
**380.5**	461.2154	449.64731	450.09076	429.23507	432.78593	431.95262	422.81015	399.03364	404.34809

**381**	455.96347	444.53144	445.1194	424.20401	428.07191	427.25223	418.15475	394.51784	400.20688
**381.5**	450.77447	439.54322	440.21818	419.24058	423.44873	422.63602	413.58544	390.08405	396.13381
**382**	445.64498	434.67059	435.39862	414.35475	418.90453	418.09932	409.09261	385.73977	392.13261
**382.5**	440.58535	429.90251	430.68333	409.55295	414.42834	413.64136	404.67501	381.49294	388.20085
**383**	435.61374	425.22826	426.08538	404.82498	410.02017	409.26447	400.33155	377.34667	384.33592
**383.5**	430.74492	420.63749	421.60131	400.16268	405.67937	404.95996	396.0565	373.30011	380.5371
**384**	425.99326	416.11161	417.20678	395.57097	401.40373	400.7117	391.8388	369.33791	376.80382
**384.5**	421.36231	411.63727	412.87451	391.06182	397.18602	396.50278	387.67284	365.44597	373.13242
**385**	416.84037	407.21384	408.57955	386.63742	393.02215	392.33116	383.5607	361.60805	369.51911
**385.5**	412.39768	402.84875	404.31384	382.29015	388.91042	388.19926	379.50919	357.81085	365.95924
**386**	408.00801	398.54411	400.08245	378.01626	384.85846	384.11297	375.51871	354.04489	362.44174
**386.5**	403.65186	394.29117	395.90443	373.81772	380.8698	380.07056	371.5828	350.32346	358.94823
**387**	399.32449	390.07968	391.7947	369.69073	376.93599	376.07605	367.6928	346.66645	355.46086
**387.5**	395.022	385.89991	387.75914	365.61138	373.03873	372.13756	363.84184	343.08372	351.96983
**388**	390.74825	381.74696	383.78714	361.55468	369.16634	368.25603	360.02535	339.56193	348.47573
**388.5**	386.49436	377.61432	379.85497	357.50998	365.30862	364.41333	356.24137	336.08369	344.98167
**389**	382.24935	373.50203	375.92876	353.48813	361.45154	360.58504	352.48679	332.6326	341.49032
**389.5**	377.99323	369.41141	371.9766	349.49458	357.5814	356.75483	348.74951	329.19662	338.00001
**390**	373.72379	365.34545	367.98512	345.51945	353.69334	352.9135	345.01474	325.75993	334.51105
**390.5**	369.44649	361.29489	363.96013	341.53901	349.78746	349.04954	341.27178	322.30776	331.01958
**391**	365.17501	357.24707	359.91733	337.53672	345.864	345.15209	337.51307	318.83135	327.52058
**391.5**	360.90757	353.18408	355.86269	333.51243	341.92079	341.22174	333.7249	315.3318	324.00456
**392**	356.63588	349.09482	351.79672	329.47325	337.96308	337.27294	329.89578	311.81286	320.46315
**392.5**	352.3428	344.9722	347.71978	325.42402	334.00055	333.32002	326.01976	308.273	316.88779
**393**	348.01608	340.82256	343.63425	321.37093	330.03786	329.36279	322.10563	304.70829	313.27526
**393.5**	343.64587	336.65618	339.53329	317.32708	326.06118	325.38678	318.16647	301.11729	309.62436
**394**	339.23643	332.48141	335.41026	313.30446	322.05372	321.3798	314.21316	297.49985	305.94084
**394.5**	334.80819	328.29405	331.25984	309.29639	318.0107	317.34485	310.24819	293.85097	302.23141
**395**	330.39687	324.08707	327.08613	305.27484	313.93847	313.28636	306.27394	290.16468	298.50822
**395.5**	326.02823	319.85634	322.88934	301.21166	309.83716	309.19956	302.29291	286.44251	294.78112

**396**	321.69813	315.60142	318.67249	297.10115	305.70989	305.07771	298.30555	282.69281	291.05785
**396.5**	317.3805	311.31966	314.43751	292.95973	301.5676	300.92909	294.30859	278.92188	287.33567
**397**	313.05122	307.00891	310.19059	288.80541	297.42553	296.77197	290.30438	275.13338	283.60894
**397.5**	308.6946	302.67584	305.93496	284.64173	293.28838	292.61444	286.29525	271.33215	279.87148
**398**	304.30187	298.33595	301.67065	280.46767	289.15397	288.45026	282.28055	267.52179	276.12238
**398.5**	299.87457	294.00407	297.394	276.29428	285.0187	284.27583	278.25765	263.70015	272.35787
**399**	295.42373	289.68322	293.10824	272.13714	280.88891	280.10393	274.22754	259.86549	268.5815
**399.5**	290.97011	285.36417	288.82462	268.00129	276.77316	275.95784	270.19204	256.02578	264.80835
**400**	286.53749	281.03646	284.54958	263.87708	272.6796	271.85011	266.15422	252.20391	261.05889
**400.5**	282.1517	276.70369	280.28237	259.75831	268.61134	267.78169	262.12369	248.42312	257.34169
**401**	277.82486	272.38305	276.02345	255.65072	264.57619	263.74747	258.12045	244.6959	253.65708
**401.5**	273.55961	268.09733	271.78377	251.56734	260.57746	259.74542	254.16484	241.01408	250.00967
**402**	269.35292	263.86124	267.57428	247.51945	256.615	255.77305	250.26355	237.36316	246.40747
**402.5**	265.2066	259.68654	263.40445	243.51167	252.68847	251.8263	246.40682	233.73427	242.85253
**403**	261.11768	255.58417	259.28168	239.5478	248.8079	247.90516	242.58284	230.13057	239.33791
**403.5**	257.08923	251.5627	255.21772	235.63745	244.97996	244.01979	238.78988	226.55527	235.86035
**404**	253.11874	247.61668	251.22059	231.7911	241.20582	240.18709	235.02965	223.01375	232.42191
**404.5**	249.20029	243.7309	247.29496	228.01042	237.48209	236.42025	231.30213	219.51706	229.03164
**405**	245.32247	239.89119	243.43404	224.28472	233.80998	232.72552	227.61437	216.0857	225.69063
**405.5**	241.48395	236.09904	239.63025	220.60866	230.19104	229.10407	223.98365	212.73391	222.39607
**406**	237.6852	232.35921	235.88456	216.99438	226.62671	225.55396	220.42374	209.4647	219.14167
**406.5**	233.92858	228.67356	232.21115	213.46261	223.11412	222.06938	216.93309	206.26587	215.92622
**407**	230.21502	225.0393	228.61903	210.02357	219.65049	218.6458	213.49927	203.12721	212.74447
**407.5**	226.55146	221.45921	225.10479	206.67309	216.23572	215.2868	210.11593	200.04274	209.59288
**408**	222.94669	217.94112	221.65522	203.39979	212.87621	212.00044	206.78864	197.01474	206.47484
**408.5**	219.41231	214.48877	218.26147	200.19354	209.57575	208.78683	203.52572	194.04043	203.41149
**409**	215.95598	211.10046	214.91588	197.0445	206.33958	205.6367	200.33116	191.11715	200.42149
**409.5**	212.5751	207.78092	211.61997	193.9432	203.17403	202.53834	197.20967	188.24527	197.51065
**410**	209.26244	204.54014	208.38217	190.88646	200.08762	199.48922	194.17144	185.43817	194.66886
**410.5**	206.017	201.38286	205.22346	187.88347	197.08165	196.49424	191.22081	182.7089	191.88817

**411**	202.84491	198.3014	202.16297	184.95363	194.15266	193.55958	188.34535	180.06236	189.16225
**411.5**	199.74875	195.28949	199.20861	182.11291	191.28954	190.68571	185.52904	177.48984	186.48832
**412**	196.72784	192.34854	196.34624	179.36325	188.48357	187.8715	182.76704	174.98145	183.86049
**412.5**	193.77838	189.4844	193.5507	176.69129	185.73288	185.11849	180.06588	172.52859	181.2795
**413**	190.89837	186.69653	190.80037	174.08427	183.0408	182.42668	177.42793	170.12943	178.75174
**413.5**	188.081	183.9821	188.08431	171.53744	180.40681	179.78839	174.8489	167.78168	176.29004
**414**	185.32319	181.33769	185.3993	169.059	177.82769	177.19544	172.32351	165.48355	173.9013
**414.5**	182.61933	178.7622	182.75092	166.6521	175.30405	174.64366	169.84853	163.23349	171.58269
**415**	179.9672	176.25299	180.15447	164.31131	172.8384	172.13348	167.42165	161.03378	169.32662
**415.5**	177.35747	173.80091	177.62547	162.02284	170.42879	169.66565	165.03754	158.88383	167.12806
**416**	174.78803	171.39487	175.16875	159.78491	168.06749	167.24642	162.69326	156.77958	164.98319
**416.5**	172.25945	169.03002	172.77616	157.6057	165.74395	164.88334	160.39373	154.71236	162.88228
**417**	169.78397	166.70948	170.43749	155.48688	163.4521	162.57623	158.1514	152.67813	160.81555
**417.5**	167.36824	164.43555	168.14303	153.41045	161.19415	160.31477	155.97481	150.67276	158.77921
**418**	165.01666	162.20384	165.88778	151.35322	158.97707	158.09008	153.86518	148.69505	156.7751
**418.5**	162.72432	160.00689	163.66663	149.30707	156.80346	155.90521	151.81442	146.74135	154.8001
**419**	160.49413	157.83909	161.47887	147.28015	154.66833	153.77159	149.80892	144.80959	152.85005
**419.5**	158.32576	155.69742	159.31861	145.27709	152.56237	151.69152	147.83265	142.89529	150.9256
**420**	156.21147	153.5818	157.17854	143.29089	150.47775	149.65085	145.87549	140.99696	149.03261
**420.5**	154.12662	151.48846	155.05034	141.31648	148.41244	147.63217	143.93347	139.11552	147.17283
**421**	152.05478	149.41231	152.93618	139.36337	146.36924	145.63151	142.00792	137.25145	145.34399
**421.5**	149.9967	147.34969	150.84072	137.44832	144.3467	143.6517	140.10017	135.40221	143.54391
**422**	147.9664	145.30623	148.76653	135.5784	142.33969	141.68587	138.21162	133.5665	141.77637
**422.5**	145.96886	143.28767	146.7079	133.74734	140.34868	139.72154	136.34287	131.74915	140.04264
**423**	144.00112	141.29728	144.66307	131.94617	138.38321	137.75746	134.49588	129.95777	138.33759
**423.5**	142.05883	139.33135	142.6383	130.17181	136.44982	135.80972	132.67118	128.19786	136.65219
**424**	140.14444	137.38872	140.64391	128.41735	134.54764	133.89308	130.86502	126.47005	134.98124
**424.5**	138.25987	135.47078	138.67884	126.67379	132.66997	132.00878	129.07348	124.77089	133.32334
**425**	136.40087	133.58441	136.73343	124.93192	130.81674	130.14461	127.29603	123.09626	131.67335
**425.5**	134.55578	131.73362	134.80353	123.19187	128.99102	128.2917	125.53652	121.43979	130.02658

**426**	132.71511	129.9176	132.8964	121.45954	127.19632	126.44789	123.79538	119.79327	128.38366
**426.5**	130.87973	128.12797	131.01911	119.74805	125.42776	124.61768	122.06644	118.14979	126.75293
**427**	129.0546	126.35812	129.1687	118.07115	123.68221	122.80305	120.34366	116.51073	125.13865
**427.5**	127.24434	124.6049	127.34074	116.43684	121.95895	121.00911	118.63137	114.88814	123.54134
**428**	125.45266	122.86774	125.53533	114.84477	120.26049	119.24371	116.94402	113.29369	121.95982
**428.5**	123.68496	121.13736	123.75794	113.28883	118.58292	117.51561	115.29264	111.73085	120.39839
**429**	121.94051	119.40543	122.01053	111.75843	116.92468	115.82374	113.67562	110.19566	118.85769
**429.5**	120.21655	117.67496	120.29205	110.24282	115.28391	114.16239	112.08791	108.68688	117.33261
**430**	118.51045	115.96132	118.59884	108.73768	113.65919	112.5242	110.52907	107.20619	115.81381
**430.5**	116.8211	114.27578	116.9306	107.24692	112.04291	110.90929	108.99754	105.7524	114.29941
**431**	115.14264	112.61963	115.28837	105.77469	110.43235	109.32013	107.48243	104.31972	112.79475
**431.5**	113.47152	110.98877	113.67234	104.31894	108.83027	107.75912	105.97234	102.90247	111.30667
**432**	111.81204	109.3823	112.07949	102.87463	107.2473	106.22485	104.46583	101.50353	109.83647
**432.5**	110.1759	107.79893	110.51016	101.43906	105.69207	104.71595	102.97243	100.13089	108.38623
**433**	108.57041	106.23595	108.96487	100.01391	104.17246	103.22859	101.50047	98.79386	106.95999
**433.5**	106.99638	104.68678	107.44449	98.60311	102.69143	101.75491	100.051	97.49317	105.56337
**434**	105.45	103.15047	105.94769	97.21441	101.25258	100.28765	98.62441	96.22457	104.19482
**434.5**	103.93268	101.63081	104.47649	95.85458	99.85422	98.82962	97.22617	94.98126	102.85095
**435**	102.44811	100.13462	103.03047	94.5295	98.48858	97.3924	95.86155	93.7607	101.53177
**435.5**	100.99769	98.66438	101.60615	93.23834	97.14295	95.98916	94.52731	92.55823	100.24464
**436**	99.57555	97.22388	100.19761	91.97478	95.81035	94.62667	93.21701	91.36992	98.99195
**436.5**	98.17836	95.81885	98.80205	90.72766	94.48995	93.30516	91.9307	90.19229	97.76899
**437**	96.80852	94.45231	97.42038	89.48835	93.18677	92.02025	90.6714	89.0295	96.56588
**437.5**	95.4672	93.11935	96.05873	88.25256	91.90474	90.76576	89.43846	87.88684	95.3793
**438**	94.15001	91.81313	94.72575	87.02545	90.64865	89.53399	88.22567	86.77036	94.21125
**438.5**	92.85412	90.53275	93.42904	85.81558	89.42191	88.31802	87.03105	85.68025	93.06417
**439**	91.58328	89.28101	92.17156	84.62976	88.22711	87.11365	85.85564	84.61366	91.93705
**439.5**	90.34415	88.05892	90.95307	83.46175	87.06099	85.91921	84.70307	83.56362	90.83015
**440**	89.139	86.86136	89.76844	82.30262	85.91597	84.73656	83.57463	82.526	89.74658
**440.5**	87.96439	85.68025	88.6096	81.15072	84.78425	83.57262	82.47241	81.49829	88.68802

**441**	86.8195	84.51343	87.46899	80.01799	83.6628	82.43764	81.39775	80.48075	87.64855
**441.5**	85.7046	83.36683	86.34455	78.91063	82.55289	81.33542	80.35331	79.47338	86.62061
**442**	84.61911	82.24691	85.2387	77.82339	81.45701	80.26068	79.33869	78.47988	85.60058
**442.5**	83.55794	81.1525	84.1532	76.74647	80.37751	79.20879	78.35061	77.50416	84.5896
**443**	82.51892	80.07677	83.08653	75.68314	79.31643	78.1833	77.38054	76.54958	83.58551
**443.5**	81.50381	79.01624	82.03277	74.64388	78.27575	77.18866	76.41875	75.6149	82.58627
**444**	80.52007	77.97464	80.98652	73.63507	77.25342	76.21949	75.45682	74.69849	81.5966
**444.5**	79.56956	76.95794	79.94737	72.65321	76.24402	75.26318	74.49456	73.79924	80.62868
**445**	78.64898	75.96689	78.92003	71.69713	75.2447	74.31331	73.53736	72.9184	79.69272
**445.5**	77.74177	74.99219	77.91094	70.77376	74.26133	73.37	72.59107	72.05604	78.78811
**446**	76.84783	74.03455	76.92376	69.89158	73.29679	72.4383	71.65927	71.20771	77.91379
**446.5**	75.96785	73.09682	75.96014	69.05155	72.34825	71.51959	70.74562	70.37084	77.06566
**447**	75.10013	72.1815	75.01781	68.24612	71.4109	70.61393	69.85432	69.54638	76.23431
**447.5**	74.23638	71.2849	74.09195	67.46559	70.48566	69.72717	68.98969	68.73716	75.4045
**448**	73.32767	70.36621	73.1335	66.61424	69.55518	68.82835	68.0906	67.9106	74.52965
**448.5**	72.43179	69.46369	72.18852	65.77955	68.63808	67.94405	67.20326	67.09629	73.66481
**449**	71.54875	68.57733	71.25703	64.96153	67.73438	67.0743	66.32767	66.29424	72.80999
**449.5**	70.67855	67.70714	70.33903	64.16018	66.84407	66.21907	65.46384	65.50444	71.9652
**450**	69.82119	66.85312	69.43451	63.3755	65.96716	65.37838	64.61176	64.7269	71.13042
**450.5**	68.97667	66.01527	68.54348	62.60748	65.10363	64.55223	63.77143	63.96161	70.30567
**451**	68.145	65.19359	67.66593	61.85613	64.2535	63.74061	62.94285	63.20858	69.49093
**451.5**	67.32616	64.38807	66.80187	61.12144	63.41676	62.94352	62.12602	62.46781	68.68622
**452**	66.52016	63.59872	65.95129	60.40342	62.59341	62.16097	61.32095	61.73929	67.89153
**452.5**	65.727	62.82554	65.11419	59.70207	61.78345	61.39295	60.52763	61.02303	67.10685
**453**	64.94668	62.06853	64.29059	59.01738	60.98689	60.63946	59.74606	60.31903	66.3322
**453.5**	64.1792	61.32769	63.48046	58.34937	60.20372	59.90051	58.97624	59.62728	65.56757
**454**	63.42456	60.60301	62.68383	57.69801	59.43394	59.1761	58.21818	58.94779	64.81296
**454.5**	62.68276	59.89451	61.90067	57.06333	58.67755	58.46622	57.47187	58.28055	64.06837
**455**	61.9538	59.20217	61.13101	56.44531	57.93455	57.77087	56.73731	57.62557	63.3338
**455.5**	61.23768	58.52599	60.37482	55.84396	57.20495	57.09006	56.0145	56.98284	62.60925

**456**	60.5344	57.86599	59.63213	55.25928	56.48874	56.42378	55.30345	56.35238	61.89472
**456.5**	59.84396	57.22215	58.90291	54.69126	55.78592	55.77203	54.60415	55.73416	61.19021
**457**	59.16636	56.59448	58.18719	54.13991	55.09649	55.13482	53.9166	55.12821	60.49572
**457.5**	58.5016	55.98298	57.48495	53.60522	54.42045	54.51215	53.2408	54.53451	59.81126
**458**	57.84967	55.38765	56.79619	53.0872	53.75781	53.904	52.57676	53.95306	59.13681
**458.5**	57.21059	54.80849	56.12092	52.58585	53.10856	53.3104	51.92447	53.38388	58.47238
**459**	56.58435	54.24549	55.45913	52.10117	52.4727	52.73132	51.28393	52.82694	57.81798
**459.5**	55.97095	53.69866	54.81083	51.63315	51.85023	52.16678	50.65514	52.28227	57.17359
**460**	55.37039	53.168	54.17601	51.1818	51.24116	51.61678	50.0381	51.74985	56.53923

	**0wks 33%RH**	**0wks 74%RH**	**6wks 4℃ 33%RH**	**6wks 37℃ 33%RH**	**6wks 4℃ 74%RH**	**6wks 37℃ 74%RH**	**10wks 4℃ 33%RH**	**10wks 37℃ 33%RH**	**10wks 4℃ 74%RH**	**10wks 37℃ 74%RH**
**300**	440.46478	397.85801	425.00499	351.65536	518.19958	468.67357	506.01388	387.60091	444.43201	413.63838
**300.5**	435.20545	393.59955	419.92788	348.49169	510.64465	462.25404	498.48291	383.32856	438.52711	408.53281
**301**	429.94612	389.34109	414.85077	345.32802	503.08971	455.83451	490.95194	379.05621	432.62221	403.42725
**301.5**	424.6868	385.08262	409.77366	342.16434	495.53478	449.41498	483.42097	374.78386	426.71732	398.32168
**302**	419.42747	380.82416	404.69655	339.00067	487.97985	442.99545	475.89	370.51151	420.81242	393.21611
**302.5**	414.16814	376.5657	399.61944	335.837	480.42492	436.57592	468.35903	366.23916	414.90753	388.11055
**303**	408.90881	372.30724	394.54234	332.67332	472.86998	430.15639	460.82806	361.96681	409.00263	383.00498
**303.5**	403.64948	368.04878	389.46523	329.50965	465.31505	423.73686	453.29709	357.69446	403.09773	377.89941
**304**	398.39015	363.79032	384.38812	326.34598	457.76012	417.31732	445.76612	353.42211	397.19284	372.79385
**304.5**	393.13082	359.53186	379.31101	323.1823	450.20519	410.89779	438.23514	349.14976	391.28794	367.68828
**305**	387.8715	355.27339	374.2339	320.01863	442.65026	404.47826	430.70417	344.87741	385.38305	362.58271
**305.5**	382.61217	351.01493	369.15679	316.85496	435.09532	398.05873	423.1732	340.60506	379.47815	357.47715
**306**	377.35284	346.75647	364.07968	313.69128	427.54039	391.6392	415.64223	336.33271	373.57325	352.37158
**306.5**	372.09351	342.49801	359.00257	310.52761	419.98546	385.21967	408.11126	332.06036	367.66836	347.26601
**307**	366.83418	338.23955	353.92546	307.36394	412.43053	378.80014	400.58029	327.78801	361.76346	342.16045
**307.5**	361.57485	333.98109	348.84835	304.20026	404.8756	372.38061	393.04932	323.51566	355.85857	337.05488
**308**	356.31553	329.72262	343.77124	301.03659	397.32066	365.96108	385.51835	319.24331	349.95367	331.94931
**308.5**	351.0562	325.46416	338.69413	297.87292	389.76573	359.54154	377.98738	314.97096	344.04878	326.84375
**309**	345.79687	321.2057	333.61702	294.70924	382.2108	353.12201	370.45641	310.69861	338.14388	321.73818
**309.5**	340.53754	316.94724	328.53991	291.54557	374.65587	346.70248	362.92543	306.42626	332.23898	316.63261
**310**	335.27821	312.68878	323.4628	288.3819	367.10094	340.28295	355.39446	302.15391	326.33409	311.52705
**310.5**	330.01888	308.43032	318.38569	285.21822	359.546	333.86342	347.86349	297.88156	320.42919	306.42148
**311**	324.75955	304.17186	313.30858	282.05455	351.99107	327.44389	340.33252	293.60921	314.5243	301.31592
**311.5**	319.50023	299.91339	308.23147	278.89088	344.43614	321.02436	332.80155	289.33686	308.6194	296.21035
**312**	314.2409	295.65493	303.15436	275.7272	336.88121	314.60483	325.27058	285.06451	302.7145	291.10478
**312.5**	308.98157	291.39647	298.07725	272.56353	329.32627	308.18529	317.73961	280.79216	296.80961	285.99922
**313**	311.46196	294.26373	300.51196	275.67765	330.78627	310.00647	318.98196	283.47294	298.75392	285.66078

**313.5**	312.82353	296.05451	301.81627	278.01314	330.64647	310.39824	318.66216	285.27627	299.42431	284.33176
**314**	312.99745	296.72549	301.95451	279.48471	328.99706	309.46059	316.8849	286.02667	298.8198	282.13843
**314.5**	312.05588	296.43039	301.04078	280.13314	326.09784	307.41961	313.92059	285.73902	297.13706	279.31882
**315**	310.15882	295.38529	299.2298	280.06275	322.10137	304.47373	309.95353	284.5902	294.49667	275.92275
**315.5**	307.58686	293.8098	296.81765	279.45157	317.30039	300.81961	305.31196	282.86275	291.13922	272.21608
**316**	304.49412	291.73549	293.8902	278.35294	311.88882	296.51765	300.09529	280.64	287.14725	268.42
**316.5**	301.13745	289.33275	290.68412	276.97373	306.25412	291.84804	294.53216	278.10431	282.87647	264.97137
**317**	297.76902	286.83706	287.39824	275.56922	300.5798	287.07745	288.74608	275.48902	278.59275	262.26529
**317.5**	294.83196	284.70314	284.50176	274.52843	295.38961	282.77275	283.3751	273.1902	274.7749	260.68922
**318**	292.75706	283.36275	282.36275	274.13353	291.12569	279.32196	278.96608	271.53627	271.69804	260.35608
**318.5**	291.94588	283.16863	281.42627	274.69157	288.32314	277.18059	276.06059	270.87529	269.83235	261.33588
**319**	292.48157	284.2298	281.87451	276.35333	287.04725	276.47804	274.62176	271.36098	269.33333	263.55686
**319.5**	294.48667	286.60098	283.85157	279.23745	287.43882	277.41039	274.80961	273.15588	270.40216	267.02706
**320**	297.86627	290.19529	287.14784	283.26118	289.4649	279.80745	276.52667	276.22706	272.83314	271.58216
**320.5**	302.56588	294.9549	291.63941	288.35961	293.12784	283.55314	279.85706	280.5298	276.54275	277.13824
**321**	308.33627	300.74412	297.12451	294.39608	298.08451	288.36588	284.42647	285.82196	281.25	283.5702
**321.5**	315.13294	307.52549	303.6102	301.3102	304.11529	294.22451	290.1098	292.02471	286.98431	290.79529
**322**	322.82608	315.17706	311.01176	309.0002	311.05784	301.03608	296.66078	299.05098	293.61608	298.63039
**322.5**	331.35784	323.54549	319.21216	317.39392	318.83275	308.67647	304.05235	306.85098	301.07863	306.88
**323**	340.49843	332.45392	327.98353	326.33588	327.31039	316.91804	312.12275	315.19745	309.13882	315.38196
**323.5**	350.07569	341.76569	337.13706	335.65098	336.27196	325.59824	320.75686	323.90333	317.6302	324.04255
**324**	359.92	351.37333	346.57569	345.19804	345.5798	334.60706	329.74922	332.84706	326.38961	332.81667
**324.5**	369.92157	361.14451	356.19745	354.8698	355.12255	343.84627	338.90824	341.96314	335.27706	341.64118
**325**	379.99608	370.96294	365.9049	364.58706	364.80176	353.19098	348.1051	351.18157	344.21941	350.46039
**325.5**	390.12765	380.77765	375.61961	374.29647	374.51569	362.55882	357.32333	360.43451	353.1798	359.24647
**326**	400.29078	390.60667	385.32451	383.99314	384.19098	371.92078	366.57608	369.65804	362.13314	367.98196
**326.5**	410.42745	400.43294	394.9949	393.65765	393.79941	381.26588	375.86235	378.80922	371.0349	376.63608
**327**	420.49784	410.22275	404.61255	403.30588	403.38275	390.57039	385.15902	387.8902	379.91412	385.19784
**327.5**	430.47118	419.94098	414.1698	412.90255	412.96902	399.81373	394.43451	396.92471	388.77922	393.71255
**328**	440.39196	429.62725	423.72608	422.45314	422.56667	409.00549	403.66059	405.89373	397.61882	402.21216
**328.5**	450.26608	439.30549	433.27765	431.92608	432.11098	418.1351	412.80706	414.76608	406.34784	410.68471
**329**	460.1302	448.94078	442.81275	441.36176	441.55196	427.21118	421.88235	423.53078	414.94176	419.0849
**329.5**	469.97529	458.46706	452.26725	450.7502	450.86922	436.23353	430.89431	432.23686	423.41	427.39529
**330**	479.81882	467.8851	461.63529	460.0702	460.12157	445.22353	439.86	440.89608	431.82137	435.61333
**330.5**	489.60608	477.24255	470.89706	469.25843	469.33804	454.17	448.75255	449.47922	440.18725	443.75255
**331**	499.29804	486.56725	480.0751	478.34333	478.52118	463.04549	457.56922	457.9198	448.49255	451.7949
**331.5**	508.87706	495.82118	489.15922	487.35353	487.61588	471.79706	466.29	466.22098	456.69922	459.70843
**332**	518.33608	504.93686	498.13333	496.3049	496.57647	480.40235	474.91784	474.40353	464.77529	467.44118
**332.5**	527.62529	513.87451	506.91608	505.11804	505.36235	488.84392	483.42706	482.47941	472.67706	474.97745
**333**	536.66255	522.61902	515.44843	513.6998	513.95	497.10196	491.77078	490.42529	480.37314	482.33549
**333.5**	545.4698	531.17314	523.76353	522.01784	522.32961	505.16235	499.90392	498.18314	487.87098	489.50059
**334**	554.09412	539.52941	531.91706	530.12078	530.50667	513.02275	507.79235	505.69275	495.19569	496.43157
**334.5**	562.55863	547.6751	539.90373	538.06039	538.45333	520.70412	515.44784	512.92137	502.32314	503.09843
**335**	570.76902	555.58784	547.65216	545.81118	546.12275	528.19647	522.86863	519.89706	509.19725	509.5198

**335.5**	578.68549	563.24863	555.14	553.31765	553.49176	535.46314	530.08	526.63569	515.80059	515.70098
**336**	586.2898	570.61843	562.35706	560.49765	560.55078	542.41373	537.02824	533.07176	522.12784	521.61078
**336.5**	593.59902	577.6849	569.28196	567.35529	567.32765	549.00294	543.69902	539.16196	528.18	527.23176
**337**	600.56	584.4249	575.83843	573.89843	573.79608	555.23667	550.06176	544.9198	533.93882	532.58529
**337.5**	607.20451	590.82412	582.02784	580.19647	579.96706	561.20471	556.12804	550.44765	539.40039	537.69725
**338**	613.57922	596.84804	587.90725	586.21529	585.82353	566.95176	561.88745	555.71157	544.57098	542.54098
**338.5**	619.72196	602.54549	593.57118	591.94902	591.4298	572.47078	567.37275	560.68216	549.48431	547.11431
**339**	625.55725	607.97235	598.98157	597.36216	596.77235	577.71078	572.59804	565.31392	554.16627	551.40765
**339.5**	631.05588	613.18294	604.09176	602.51176	601.86941	582.66922	577.58667	569.68627	558.61784	555.43255
**340**	636.24647	618.11784	608.87196	607.38373	606.65196	587.34824	582.27882	573.78902	562.80059	559.18745
**340.5**	641.19098	622.73529	613.39333	611.99549	611.13451	591.74961	586.65725	577.63373	566.69275	562.69216
**341**	645.83863	627.00471	617.66	616.2949	615.29922	595.85882	590.72118	581.17941	570.28157	565.94059
**341.5**	650.1198	630.95039	621.6402	620.27843	619.17216	599.68275	594.49902	584.44588	573.55686	568.91137
**342**	654.02078	634.60706	625.29882	623.93961	622.74647	603.22902	597.99333	587.43608	576.52255	571.5951
**342.5**	657.60804	638.01647	628.69451	627.32941	626.05588	606.49294	601.22451	590.17118	579.19647	574.04745
**343**	660.90647	641.16902	631.84608	630.44294	629.09431	609.47745	604.18412	592.64569	581.58725	576.2998
**343.5**	663.90706	644.04549	634.74235	633.27314	631.85804	612.22608	606.88157	594.84569	583.69039	578.34784
**344**	666.59275	646.65706	637.33333	635.81765	634.31745	614.75471	609.34667	596.76627	585.51569	580.17098
**344.5**	669.01745	649.00549	639.62608	638.10863	636.49314	617.0498	611.58627	598.42863	587.09608	581.77824
**345**	671.21157	651.08745	641.63863	640.1502	638.43412	619.0851	613.60961	599.85078	588.45569	583.13961
**345.5**	673.19941	652.90922	643.42549	641.94216	640.16137	620.88706	615.4002	601.02647	589.60608	584.24902
**346**	674.94686	654.50392	645.01353	643.51373	641.63216	622.46784	616.96902	601.97824	590.55765	585.1349
**346.5**	676.45725	655.88412	646.39941	644.90078	642.8049	623.82294	618.3102	602.73039	591.30314	585.83804
**347**	677.70373	656.99922	647.53275	646.09255	643.71098	624.90392	619.41863	603.27608	591.83804	586.3551
**347.5**	678.72804	657.84843	648.38314	647.04725	644.42137	625.73039	620.28431	603.58471	592.15745	586.67235
**348**	679.52157	658.46294	648.98431	647.73059	644.97137	626.34	620.90961	603.65275	592.27529	586.82196
**348.5**	680.1049	658.88451	649.38353	648.15373	645.30745	626.77157	621.2798	603.5051	592.17843	586.81627
**349**	680.4449	659.09686	649.62059	648.35	645.42294	626.99157	621.40804	603.16902	591.87118	586.62843
**349.5**	680.5749	659.09588	649.68765	648.35333	645.34255	626.9849	621.36627	602.66294	591.37804	586.24098
**350**	680.48824	658.88196	649.58608	648.18765	645.08784	626.78098	621.23784	601.98961	590.75941	585.69843
**350.5**	680.19373	658.49216	649.28353	647.86667	644.62882	626.42176	621.03529	601.14667	590	585.0351
**351**	679.66706	657.93686	648.79373	647.39118	643.98	625.92333	620.69686	600.13686	589.09725	584.19667
**351.5**	678.95745	657.21706	648.12333	646.74118	643.18902	625.28549	620.13824	598.98078	588.02471	583.16412
**352**	678.11706	656.32176	647.30373	645.91392	642.27431	624.51059	619.33431	597.68078	586.8198	581.96784
**352.5**	677.1551	655.23804	646.33059	644.89647	641.17961	623.57569	618.32333	596.2102	585.43529	580.68824
**353**	676.03863	653.97333	645.18588	643.70863	639.85235	622.43824	617.15059	594.5502	583.86627	579.27275
**353.5**	674.7102	652.51549	643.80412	642.34137	638.2951	621.11471	615.83078	592.73549	582.11471	577.68431
**354**	673.14765	650.84392	642.1902	640.79608	636.56059	619.64235	614.35373	590.81569	580.25843	575.89745
**354.5**	671.36039	648.96804	640.36608	639.04098	634.6851	618.02196	612.70431	588.80686	578.29216	573.99922
**355**	669.38647	646.95078	638.38706	637.09843	632.68255	616.18647	610.91216	586.63627	576.19098	571.97314
**355.5**	667.23235	644.83294	636.26588	634.98824	630.50882	614.12706	608.98784	584.2549	573.90059	569.80608
**356**	664.88569	642.57961	633.99824	632.73843	628.12863	611.87176	606.91667	581.67137	571.42922	567.44235
**356.5**	662.33216	640.10863	631.54255	630.31765	625.52843	609.48647	604.63451	578.95941	568.80157	564.90784
**357**	659.57686	637.39569	628.89176	627.72255	622.76078	606.98863	602.14922	576.15118	566.01745	562.20078

**357.5**	656.67784	634.49	626.07196	624.96098	619.88314	604.35451	599.46549	573.22314	563.08137	559.37216
**358**	653.67314	631.44451	623.17137	622.08216	616.93647	601.5598	596.62863	570.13451	560.02431	556.42176
**358.5**	650.59608	628.26176	620.2102	619.08333	613.89098	598.59686	593.65863	566.86392	556.89451	553.36588
**359**	647.3798	624.93902	617.11725	615.94549	610.70431	595.49745	590.60549	563.4398	553.69471	550.19725
**359.5**	643.97588	621.46098	613.83	612.62451	607.36	592.29863	587.45549	559.92078	550.37922	546.94686
**360**	640.34784	617.85392	610.3649	609.12039	603.87961	588.99824	584.19333	556.35078	546.93804	543.63627
**360.5**	636.56176	614.13529	606.78392	605.45314	600.27333	585.56216	580.81412	552.69843	543.3949	540.2498
**361**	632.65569	610.33902	603.11039	601.66176	596.55275	581.97078	577.34549	548.94098	539.78647	536.76059
**361.5**	628.68686	606.44196	599.35	597.78922	592.72471	578.26745	573.80078	545.12275	536.11353	533.16078
**362**	624.65569	602.42549	595.50471	593.87843	588.79647	574.50824	570.17588	541.27686	532.37118	529.48196
**362.5**	620.54255	598.27078	591.61686	589.9298	584.79412	570.72824	566.44667	537.38333	528.52647	525.72412
**363**	616.28333	594.04137	587.66451	585.90431	580.74039	566.86314	562.62412	533.36451	524.56529	521.89471
**363.5**	611.84667	589.77608	583.58804	581.78353	576.64412	562.87961	558.72235	529.22804	520.50529	518.0002
**364**	607.28039	585.45588	579.35314	577.5802	572.44373	558.78333	554.77529	525.01824	516.39529	514.07706
**364.5**	602.66176	581.02706	575.02961	573.30686	568.12333	554.65216	550.7698	520.7902	512.24804	510.14353
**365**	598.02471	576.49824	570.67725	568.93882	563.71157	550.49765	546.66549	516.52882	508.05353	506.17196
**365.5**	593.33725	571.93627	566.30118	564.46882	559.28157	546.28431	542.4498	512.22176	503.81373	502.12529
**366**	588.59843	567.37961	561.83157	559.93647	554.82255	541.9649	538.18176	507.84569	499.53569	497.98353
**366.5**	583.84275	562.79098	557.26882	555.40471	550.31039	537.57157	533.90941	503.44667	495.2451	493.80176
**367**	579.08765	558.13333	552.6649	550.87667	545.73137	533.16176	529.64333	499.03294	490.95431	489.63137
**367.5**	574.29529	553.40569	548.06941	546.32098	541.11706	528.75137	525.36196	494.61549	486.65922	485.50902
**368**	569.44294	548.64373	543.46216	541.71314	536.49471	524.34059	521.06431	490.17745	482.35196	481.37549
**368.5**	564.58647	543.8651	538.82588	537.08784	531.88647	519.90961	516.77353	485.74235	478.04863	477.21039
**369**	559.78157	539.07078	534.16353	532.46333	527.28157	515.41863	512.46843	481.29431	473.76137	472.99059
**369.5**	555.03392	534.24922	529.49706	527.82118	522.68863	510.84549	508.11059	476.81196	469.50451	468.74941
**370**	550.27137	529.41078	524.8351	523.13863	518.08392	506.23471	503.69431	472.28098	465.24667	464.48294
**370.5**	545.45412	524.56039	520.17059	518.44078	513.45745	501.67569	499.26431	467.73882	460.98275	460.23667
**371**	540.57882	519.69882	515.5	513.74431	508.78118	497.17137	494.81627	463.23412	456.69098	456.00275
**371.5**	535.69647	514.83745	510.79451	509.02745	504.08314	492.68392	490.34686	458.77686	452.37333	451.77118
**372**	530.80686	509.98471	506.05039	504.2702	499.39098	488.15451	485.84039	454.34745	448.02059	447.50529
**372.5**	525.90196	505.17137	501.29078	499.50569	494.73078	483.61294	481.33961	449.8902	443.63804	443.22
**373**	520.92588	500.37667	496.53863	494.76137	490.06471	479.04412	476.85745	445.3802	439.22647	438.92098
**373.5**	515.89902	495.59	491.79098	490.02118	485.38882	474.47039	472.40863	440.8502	434.81098	434.61961
**374**	510.85216	490.8051	487.04588	485.25059	480.73431	469.9049	467.96588	436.34824	430.43118	430.33863
**374.5**	505.84059	486.03529	482.28608	480.47078	476.11196	465.36922	463.51471	431.88078	426.08353	426.09647
**375**	500.84686	481.24333	477.47569	475.69824	471.47843	460.82392	459.02725	427.41471	421.75098	421.86804
**375.5**	495.86549	476.40451	472.61275	470.92745	466.79196	456.22922	454.50431	422.93706	417.39902	417.57784
**376**	490.87745	471.53176	467.75255	466.14118	462.07294	451.60627	449.96804	418.46059	413.05196	413.22961
**376.5**	485.9098	466.66216	462.93098	461.35725	457.34647	446.98176	445.43373	413.97235	408.69471	408.89882
**377**	480.94922	461.78706	458.13745	456.58157	452.61647	442.36275	440.92843	409.4651	404.32451	404.64549
**377.5**	475.97745	456.8751	453.32235	451.79745	447.89706	437.6998	436.42529	404.94549	399.91549	400.43314
**378**	470.96667	451.94529	448.45902	446.9902	443.19431	433.01608	431.88804	400.47216	395.5202	396.18471
**378.5**	465.93706	447.01451	443.55078	442.16294	438.4751	428.34275	427.28922	396.02627	391.15451	391.88961
**379**	460.91686	442.09196	438.65314	437.32706	433.7049	423.70137	422.67529	391.59451	386.81725	387.58118

**379.5**	455.92765	437.16235	433.78784	432.48843	428.8851	419.05137	418.07392	387.12627	382.45431	383.29824
**380**	450.9198	432.25118	428.9451	427.64882	424.08059	414.39549	413.48745	382.64412	378.09902	378.99765
**380.5**	445.86078	427.32608	424.08588	422.81333	419.31118	409.76333	408.86549	378.15431	373.77196	374.68961
**381**	440.74667	422.35471	419.22784	417.99431	414.58176	405.17961	404.20863	373.69549	369.46863	370.38039
**381.5**	435.64784	417.3351	414.39882	413.21863	409.8751	400.61765	399.55784	369.27961	365.13686	366.12118
**382**	430.61373	412.36529	409.61667	408.49137	405.19686	396.03627	394.96392	364.91373	360.78922	361.89431
**382.5**	425.66255	407.49039	404.85863	403.77902	400.52647	391.42725	390.42765	360.55843	356.48745	357.7051
**383**	420.72373	402.68608	400.11745	399.05745	395.87353	386.84569	385.95451	356.19549	352.26961	353.5349
**383.5**	415.76882	397.86412	395.37196	394.32216	391.20765	382.33588	381.51667	351.82843	348.10745	349.40667
**384**	410.8	393.01765	390.63667	389.60549	386.52059	377.90412	377.08333	347.49294	343.94373	345.30294
**384.5**	405.88118	388.17314	385.94216	384.92196	381.82588	373.49353	372.61118	343.20059	339.76039	341.22118
**385**	401.02588	383.39529	381.29392	380.30627	377.18353	369.0602	368.13824	338.93804	335.57431	337.12922
**385.5**	396.24118	378.65784	376.68059	375.73647	372.62333	364.60314	363.70961	334.68118	331.42157	333.02549
**386**	391.48392	373.94784	372.07824	371.19922	368.14353	360.1551	359.33627	330.44176	327.32216	328.93176
**386.5**	386.73804	369.23294	367.50118	366.67706	363.68529	355.73549	354.9849	326.22922	323.26451	324.90902
**387**	381.96784	364.56431	362.94882	362.21784	359.25294	351.37353	350.66569	322.06039	319.23961	320.97471
**387.5**	377.22	359.96392	358.42882	357.81863	354.84647	347.07176	346.38118	317.95157	315.24529	317.09667
**388**	372.5502	355.45098	353.92333	353.45039	350.47667	342.82314	342.12176	313.91471	311.29059	313.22176
**388.5**	367.98196	350.98667	349.4649	349.0698	346.10196	338.61255	337.8802	309.93	307.37235	309.34255
**389**	363.46	346.54882	345.08608	344.70039	341.73569	334.42882	333.67078	305.97784	303.48235	305.48294
**389.5**	358.95431	342.13216	340.78176	340.36294	337.41863	330.26118	329.4998	302.04843	299.60294	301.65745
**390**	354.47745	337.76804	336.51784	336.07	333.17961	326.09059	325.36706	298.14725	295.74725	297.84392
**390.5**	350.05627	333.46922	332.2598	331.81961	328.98451	321.91196	321.29118	294.25647	291.93647	294.06
**391**	345.66667	329.21216	328.01157	327.62392	324.7949	317.7551	317.2851	290.37569	288.16804	290.32706
**391.5**	341.28529	324.95922	323.79902	323.47843	320.62824	313.68333	313.33725	286.5302	284.42941	286.67471
**392**	336.90569	320.72	319.64941	319.3749	316.51255	309.70549	309.4002	282.7398	280.71686	283.05745
**392.5**	332.57725	316.52275	315.54686	315.31137	312.46	305.79451	305.45294	279.01353	277.05196	279.46412
**393**	328.33275	312.39255	311.45196	311.27137	308.45314	301.90118	301.4749	275.32902	273.44059	275.8802
**393.5**	324.17412	308.30804	307.34882	307.25216	304.47627	298.02059	297.49275	271.66941	269.85804	272.34804
**394**	320.01902	304.2598	303.27549	303.24667	300.50804	294.15941	293.54529	268.02471	266.28392	268.85196
**394.5**	315.82373	300.23588	299.27275	299.26941	296.56255	290.3502	289.68647	264.42569	262.73216	265.40137
**395**	311.58941	296.23784	295.33745	295.31784	292.66471	286.59784	285.90784	260.88608	259.20098	261.97451
**395.5**	307.38059	292.2549	291.43118	291.3998	288.83196	282.88549	282.1798	257.40392	255.68941	258.57745
**396**	303.22941	288.29098	287.5449	287.5149	285.04373	279.16412	278.46392	253.95392	252.18863	255.19
**396.5**	299.14196	284.34333	283.67843	283.67804	281.28255	275.42373	274.77922	250.50412	248.73137	251.82275
**397**	295.11078	280.42078	279.85843	279.88902	277.55588	271.67314	271.12294	247.04275	245.32706	248.49255
**397.5**	291.13667	276.54451	276.0902	276.14431	273.86725	267.93961	267.49353	243.58765	241.98686	245.22
**398**	287.21686	272.7251	272.36941	272.42745	270.19569	264.24725	263.8802	240.19431	238.68196	241.98373
**398.5**	283.3502	268.95275	268.67706	268.73196	266.52059	260.62922	260.31745	236.87686	235.40176	238.75941
**399**	279.52706	265.20588	265.00686	265.06863	262.83588	257.07804	256.80451	233.60373	232.12373	235.53059
**399.5**	275.72804	261.46725	261.3551	261.4602	259.14667	253.56	253.33196	230.33	228.84843	232.29333
**400**	271.9349	257.73078	257.72647	257.88902	255.46843	250.06725	249.87314	227.0549	225.59941	229.05667
**400.5**	268.15216	254.0251	254.13824	254.35059	251.81961	246.62647	246.43706	223.79078	222.38706	225.83608
**401**	264.40529	250.38275	250.59275	250.85059	248.22569	243.24667	243.0202	220.55118	219.20078	222.67098

**401.5**	260.71451	246.80255	247.08275	247.38745	244.70961	239.89549	239.60686	217.33275	216.01137	219.53686
**402**	257.05333	243.24431	243.59078	243.93294	241.27098	236.55176	236.19549	214.14275	212.85667	216.42
**402.5**	253.39412	239.69255	240.12373	240.48725	237.89725	233.22804	232.80235	210.98765	209.7751	213.3202
**403**	249.7398	236.18373	236.68059	237.08412	234.53922	229.95098	229.46882	207.86961	206.76216	210.28157
**403.5**	246.12039	232.74471	233.28706	233.72314	231.17098	226.71392	226.20353	204.77216	203.76392	207.30471
**404**	242.54392	229.36529	229.95824	230.39176	227.81412	223.49667	223.00314	201.69824	200.77098	204.37157
**404.5**	238.99863	226.00824	226.68843	227.08157	224.51765	220.27157	219.82882	198.66314	197.81588	201.45392
**405**	235.49216	222.66059	223.44882	223.83412	221.30255	217.05275	216.66196	195.68118	194.91471	198.54706
**405.5**	232.04745	219.33137	220.22333	220.65098	218.12549	213.85627	213.50706	192.75275	192.03392	195.6751
**406**	228.68255	216.05804	217.0302	217.50902	214.94608	210.70725	210.38686	189.87275	189.16745	192.84667
**406.5**	225.38608	212.86353	213.87588	214.39431	211.75667	207.60686	207.32078	187.0402	186.34824	190.07098
**407**	222.13176	209.72882	210.76765	211.31686	208.59431	204.57451	204.31078	184.23549	183.60235	187.31941
**407.5**	218.89824	206.61647	207.68667	208.28627	205.48176	201.60863	201.3551	181.45196	180.90686	184.60118
**408**	215.67804	203.51314	204.64059	205.29196	202.42627	198.69627	198.43216	178.68824	178.23647	181.92765
**408.5**	212.49745	200.43255	201.61922	202.32137	199.40569	195.80059	195.53451	175.96294	175.58353	179.3002
**409**	209.37549	197.40294	198.63196	199.37098	196.41804	192.92725	192.66706	173.2849	172.96353	176.69569
**409.5**	206.30765	194.43961	195.68255	196.47373	193.48333	190.10412	189.84627	170.65078	170.39314	174.10549
**410**	203.25157	191.54745	192.77412	193.64706	190.61922	187.34922	187.0702	168.05392	167.86059	171.54941
**410.5**	200.21098	188.6949	189.91824	190.88333	187.81098	184.63706	184.32784	165.50059	165.35098	169.05667
**411**	197.21529	185.87588	187.11745	188.16647	185.03706	181.95667	181.61373	163.00824	162.84667	166.63706
**411.5**	194.30333	183.08275	184.3798	185.49333	182.2949	179.32118	178.92765	160.57078	160.36412	164.27196
**412**	191.46314	180.34176	181.69392	182.86039	179.61	176.75353	176.27039	158.18882	157.93137	161.92431
**412.5**	188.68196	177.64706	179.05588	180.2449	176.96137	174.23353	173.64725	155.8449	155.56118	159.58392
**413**	185.93353	175.00608	176.46137	177.63686	174.35412	171.73627	171.06863	153.51686	153.22588	157.26863
**413.5**	183.21569	172.42529	173.91647	175.07078	171.78804	169.25059	168.55196	151.18824	150.92608	155.01745
**414**	180.5298	169.90157	171.4202	172.57549	169.30039	166.80549	166.10314	148.88471	148.68784	152.83647
**414.5**	177.91	167.42804	168.96216	170.14765	166.86451	164.41784	163.71961	146.64451	146.52235	150.71667
**415**	175.35471	165.00902	166.54431	167.76902	164.46	162.08824	161.38686	144.47225	144.4051	148.63255
**415.5**	172.84843	162.65373	164.16255	165.43157	162.07137	159.80353	159.09314	142.34892	142.30441	146.5749
**416**	170.37353	160.33353	161.82882	163.14098	159.73235	157.57333	156.83471	140.26937	140.22676	144.53804
**416.5**	167.95314	158.03863	159.54765	160.89941	157.46196	155.40647	154.61235	138.2448	138.20043	142.53467
**417**	165.59647	155.78451	157.31843	158.69059	155.2649	153.27	152.41451	136.25931	136.23753	140.55922
**417.5**	163.29961	153.59725	155.12078	156.50647	153.12157	151.13118	150.24294	134.29227	134.32384	138.61684
**418**	161.04824	151.46	152.94843	154.36176	151.00255	148.98451	148.11235	132.33969	132.42576	136.7058
**418.5**	158.84431	149.3498	150.8202	152.26333	148.89608	146.87824	146.05373	130.4121	130.52875	134.84327
**419**	156.68059	147.25902	148.76275	150.20686	146.81157	144.83471	144.06367	128.51984	128.6508	133.0152
**419.5**	154.56843	145.20784	146.76667	148.19588	144.76353	142.86063	142.11759	126.66967	126.81692	131.21908
**420**	152.50706	143.20392	144.80824	146.24686	142.75912	140.92855	140.17925	124.85063	125.03339	129.43592
**420.5**	150.49627	141.25143	142.86843	144.36098	140.7892	139.02973	138.25004	123.05125	123.30376	127.67555
**421**	148.52059	139.33329	140.96341	142.51647	138.84982	137.15092	136.34129	121.273	121.62216	125.9429
**421.5**	146.56627	137.44551	139.09537	140.68843	136.94447	135.30053	134.47575	119.53749	119.98076	124.24863
**422**	144.62529	135.59204	137.26455	138.87029	135.07425	133.48382	132.65188	117.8588	118.36578	122.5898
**422.5**	142.69378	133.78276	135.44576	137.05775	133.22902	131.69967	130.86884	116.22686	116.77718	120.95192
**423**	140.7781	131.99424	133.63624	135.25408	131.40261	129.94849	129.10675	114.61763	115.20618	119.32269

**423.5**	138.87963	130.20524	131.83051	133.47218	129.60643	128.22463	127.36108	113.02202	113.64706	117.71953
**424**	137.01727	128.42304	130.03012	131.71633	127.8572	126.52316	125.63022	111.451	112.09361	116.15512
**424.5**	135.18476	126.66627	128.25571	129.9821	126.15655	124.83853	123.92255	109.90667	110.56625	114.61425
**425**	133.39375	124.92469	126.53116	128.26551	124.48878	123.18229	122.2411	108.39329	109.07235	113.08227
**425.5**	131.63941	123.18504	124.85184	126.58104	122.84647	121.54712	120.59822	106.90116	107.61678	111.57829
**426**	129.9258	121.45559	123.19584	124.92755	121.23061	119.92325	118.98686	105.43359	106.18904	110.12892
**426.5**	128.23394	119.76757	121.5709	123.31216	119.63596	118.30514	117.40061	103.98973	104.78339	108.73075
**427**	126.55871	118.12737	119.99445	121.72667	118.0498	116.70527	115.83724	102.57073	103.4088	107.35998
**427.5**	124.89924	116.52241	118.46275	120.16253	116.45641	115.13265	114.30435	101.17094	102.05884	105.99906
**428**	123.26784	114.93259	116.94424	118.60724	114.84873	113.58114	112.79578	99.79082	100.73173	104.63845
**428.5**	121.65451	113.35667	115.42869	117.06543	113.23649	112.0562	111.29137	98.44584	99.42331	103.26588
**429**	120.04482	111.79435	113.90657	115.56488	111.65041	110.56204	109.78502	97.13347	98.13925	101.88771
**429.5**	118.44469	110.26453	112.377	114.10959	110.11908	109.10324	108.295	95.84224	96.8558	100.52522
**430**	116.87718	108.77978	110.86088	112.68159	108.63139	107.67678	106.84531	94.54031	95.55886	99.19304
**430.5**	115.34178	107.34514	109.39151	111.23802	107.1611	106.29543	105.44312	93.23118	94.25833	97.89369
**431**	113.82494	105.94169	107.96663	109.80108	105.69622	104.94853	104.07165	91.92878	92.9839	96.61316
**431.5**	112.30473	104.55314	106.55086	108.39288	104.25692	103.61078	102.70784	90.66869	91.73861	95.34753
**432**	110.79796	103.16863	105.1298	107.03059	102.85955	102.2539	101.33941	89.43845	90.51498	94.08659
**432.5**	109.31806	101.80041	103.71667	105.67976	101.50549	100.89249	99.97824	88.22541	89.31437	92.83888
**433**	107.87673	100.45651	102.34124	104.3199	100.173	99.54924	98.64145	87.009	88.13761	91.61567
**433.5**	106.45539	99.13776	101.00494	102.95255	98.85029	98.23986	97.33359	85.81455	86.97445	90.42133
**434**	105.04422	97.83398	99.70486	101.60369	97.53935	96.95551	96.03704	84.66024	85.81406	89.2538
**434.5**	103.63378	96.54473	98.42924	100.28673	96.24847	95.68653	94.74176	83.54231	84.66002	88.10196
**435**	102.2432	95.27082	97.16884	99.00773	94.98065	94.42655	93.46204	82.43224	83.51876	86.96763
**435.5**	100.89486	94.00961	95.92104	97.75886	93.72522	93.1799	92.21967	81.31849	82.40931	85.84941
**436**	99.59476	92.76143	94.69551	96.52082	92.48039	91.95657	91.01788	80.20278	81.33284	84.75251
**436.5**	98.32349	91.54145	93.4911	95.27733	91.25176	90.74765	89.84147	79.09067	80.28406	83.67365
**437**	97.05969	90.35929	92.29414	94.04122	90.04822	89.54935	88.68776	78.00239	79.24331	82.61108
**437.5**	95.80229	89.20576	91.09435	92.83892	88.86627	88.36225	87.55725	76.9502	78.20563	81.56284
**438**	94.55853	88.05969	89.91529	91.68886	87.71282	87.19712	86.435	75.93057	77.16892	80.52955
**438.5**	93.33463	86.91522	88.76867	90.57229	86.57302	86.05496	85.30845	74.92425	76.1512	79.52649
**439**	92.12329	85.77671	87.65339	89.45065	85.43588	84.93529	84.18788	73.92461	75.15573	78.54653
**439.5**	90.92275	84.66261	86.54278	88.31075	84.29743	83.83186	83.08029	72.93733	74.18049	77.57651
**440**	89.73475	83.58335	85.44324	87.17633	83.18071	82.7381	81.99271	71.96729	73.21696	76.59296
**440.5**	88.56941	82.52871	84.35318	86.06	82.09614	81.6571	80.92257	71.01057	72.26902	75.61049
**441**	87.44371	81.47147	83.28194	84.95945	81.04129	80.59857	79.86984	70.07206	71.3359	74.64918
**441.5**	86.36206	80.41659	82.22871	83.86371	80.00749	79.56325	78.82216	69.15888	70.41014	73.72486
**442**	85.30927	79.38508	81.20424	82.79135	78.99067	78.54282	77.78939	68.27173	69.48427	72.82482
**442.5**	84.25945	78.39396	80.20725	81.75702	77.98408	77.53949	76.79016	67.399	68.56847	71.94373
**443**	83.207	77.42539	79.23161	80.75631	76.98039	76.57178	75.83878	66.53714	67.67151	71.07684
**443.5**	82.15345	76.46875	78.27298	79.75718	75.98782	75.63678	74.90912	65.68488	66.80088	70.21635
**444**	81.09525	75.51163	77.33045	78.77147	75.01982	74.71824	73.97651	64.85484	65.95535	69.34141
**444.5**	80.02906	74.56176	76.40167	77.81155	74.0738	73.79741	73.03984	64.04237	65.13275	68.45502
**445**	78.96659	73.61525	75.47984	76.88241	73.14669	72.87853	72.11235	63.23012	64.32198	67.57998

**445.5**	77.93708	72.71522	74.56902	75.96073	72.23049	71.97273	71.20347	62.42222	63.51025	66.74469
**446**	76.94196	71.81518	73.67055	75.03773	71.32439	71.08627	70.31018	61.63925	62.68831	65.94445
**446.5**	75.97261	70.91514	72.78184	74.1161	70.42298	70.213	69.43414	60.88318	61.86963	65.15386
**447**	75.01496	70.01511	71.89933	73.21565	69.52847	69.35486	68.56386	60.12047	61.07327	64.38431
**447.5**	74.06929	69.11507	71.0232	72.34224	68.6439	68.51953	67.70057	59.32263	60.31504	63.63069
**448**	73.16198	68.21503	70.19185	71.48172	67.79564	67.69942	66.85852	58.59493	59.56966	62.8709
**448.5**	72.25466	67.315	69.36051	70.6212	66.94738	66.87931	66.01647	57.86723	58.82428	62.11111
**449**	71.34734	66.41496	68.52916	69.76068	66.09912	66.0592	65.17442	57.13953	58.07889	61.35133
**449.5**	70.44002	65.51492	67.69782	68.90015	65.25085	65.23909	64.33237	56.41183	57.33351	60.59154
**450**	69.53271	64.61488	66.86648	68.03963	64.40259	64.41898	63.49032	55.68413	56.58813	59.83175
**450.5**	68.62539	63.71485	66.03513	67.17911	63.55433	63.59887	62.64827	54.95643	55.84275	59.07197
**451**	67.71807	62.81481	65.20379	66.31859	62.70607	62.77877	61.80622	54.22873	55.09737	58.31218
**451.5**	66.81075	61.91477	64.37244	65.45807	61.85781	61.95866	60.96416	53.50103	54.35199	57.55239
**452**	65.90343	61.01474	63.5411	64.59755	61.00955	61.13855	60.12211	52.77334	53.60661	56.79261
**452.5**	64.99612	60.1147	62.70976	63.73703	60.16128	60.31844	59.28006	52.04564	52.86122	56.03282
**453**	64.0888	59.21466	61.87841	62.87651	59.31302	59.49833	58.43801	51.31794	52.11584	55.27303
**453.5**	63.18148	58.31463	61.04707	62.01599	58.46476	58.67822	57.59596	50.59024	51.37046	54.51325
**454**	62.27416	57.41459	60.21573	61.15547	57.6165	57.85811	56.75391	49.86254	50.62508	53.75346
**454.5**	61.36685	56.51455	59.38438	60.29495	56.76824	57.038	55.91186	49.13484	49.8797	52.99367
**455**	60.45953	55.61451	58.55304	59.43443	55.91997	56.21789	55.06981	48.40714	49.13432	52.23389
**455.5**	59.55221	54.71448	57.72169	58.57391	55.07171	55.39778	54.22776	47.67944	48.38893	51.4741
**456**	58.64489	53.81444	56.89035	57.71339	54.22345	54.57767	53.38571	46.95174	47.64355	50.71431
**456.5**	57.73757	52.9144	56.05901	56.85287	53.37519	53.75756	52.54366	46.22404	46.89817	49.95453
**457**	56.83026	52.01437	55.22766	55.99235	52.52693	52.93746	51.70161	45.49634	46.15279	49.19474
**457.5**	55.92294	51.11433	54.39632	55.13183	51.67867	52.11735	50.85956	44.76865	45.40741	48.43495
**458**	55.01562	50.01121	53.56497	54.27131	50.8304	51.29724	50.01751	44.04095	44.66203	47.67517
**458.5**	54.1083	49.15823	52.73363	53.41079	49.98214	50.47713	49.17546	43.31325	43.91665	46.91538
**459**	53.20099	48.22491	51.90229	52.55027	49.13388	49.65702	48.33341	42.58555	43.17126	46.15559
**459.5**	52.29367	47.07921	51.07094	51.68975	48.28562	48.83691	47.49136	41.85785	42.42588	45.39581
**460**	51.38635	46.12864	50.2396	50.82923	47.43736	48.0168	46.64931	41.13015	41.6805	44.63602

Table.3. Changes in PPI volume size distributions

	0 wks	6wks -20℃	6 wks 4℃	6wks 37℃	6wks 37℃ AP	10wks -20℃	10wks 4℃	10wks 37℃	10wks 37℃ AP
**0.4**	0	0	0	0	0	0	0	0	0
**0.46**	0	0	0	0	0	0	0	0	0
**0.54**	0	0	0	0	0	0	0	0	0
**0.62**	0	0	0	0	0	0	0	0	0
**0.72**	0	0	0	0	0	0	0	0	0
**0.83**	0	0	0	0	0	0	0	0	0
**0.97**	0	0	0	0	0	0	0	0	0

**1.12**	0	0	0	0	0	0	0	0	0
**1.29**	0	0	0	0	0	0	0	0	0
**1.5**	0	0	0	0	0	0	0	0	0
**1.74**	4.09	0	0	0	0	0	0	0	0
**2.01**	15.2	0	0	0	0	0	0	0	0
**2.33**	23.2	0	0	0	0	0	0	0	0
**2.7**	19.8	0	0	0	0	0	0	0	0
**3.12**	11.4	0	0	0	0	0	0	0	0
**3.62**	5.63	0	0	0	0	0	0	0	0
**4.19**	3.18	6.29	0	0	0	0	0	0	0
**4.85**	2.19	19.6	0.106	0	0	0	0	0	0
**5.61**	1.6	24.4	6.05	0	0	0	0	0	0
**6.5**	1.32	16.4	19	5.66	0	0	0	0	0
**7.53**	1.16	7.52	25.4	23.8	4.35	0.528	0	0	0
**8.72**	0.96	3.87	20.2	30.9	16	14	3.7	0	0
**10.1**	0.83	0.97	11.7	25.1	25.2	36.1	16.8	0	0
**11.7**	0.54	0.62	5.77	6.37	23.3	34	30.5	4.12	0
**13.5**	0.31	0.47	3.07	0.0646	14.9	13.2	28.7	18.3	6.76
**15.7**	0.16	0.35	2.27	0	7.34	1.79	15.1	31.9	22.8
**18.2**	0.0697	0.25	2.01	0	3.25	0	4.27	28	31.4
**21**	0.0364	0.13	1.67	0	1.72	0	0.459	12.8	23.6
**24.4**	0.0201	0.098	1.17	0.0845	1.23	0	0	2.55	10.7
**28.2**	0.0188	0.061	0.66	0.355	0.902	0	0	0	2.75
**32.7**	0	0.0599	0.283	0.578	0.547	0	0	0	0.293
**37.8**	0	0.0549	0.0813	0.453	0.247	0	0	0	0.0609
**43.8**	0	0.0474	0.0115	0.169	0.0731	0	0	0	0.226
**50.7**	0	0.0323	0	0.0233	0.0106	0	0	0.0638	0.354
**58.8**	0	0.0151	0	0	0.00609	0	0	0.249	0.31
**68.1**	0	0.00402	0	0	0.0262	0	0	0.423	0.164
**78.8**	0	0.00182	0	0	0.0526	0	0	0.429	0.0493

**91.3**	0.00699	0.00651	0.00753	0	0.0699	0.00353	0	0.298	0.0187
**106**	0.00364	0.0141	0.0315	0.0114	0.0731	0.0227	0.00206	0.146	0.0457
**122**	0.00828	0.0212	0.0622	0.0529	0.0671	0.0549	0.0207	0.0466	0.0813
**142**	0.0126	0.026	0.0829	0.106	0.0589	0.0721	0.0612	0.00738	0.0955
**164**	0.0152	0.028	0.0879	0.123	0.053	0.0614	0.0924	0.00123	0.0846
**190**	0.016	0.0282	0.0802	0.0906	0.0508	0.0371	0.0887	0.00884	0.0603
**220**	0.0159	0.0275	0.0653	0.0404	0.0518	0.015	0.0583	0.0264	0.0361
**255**	0.0156	0.0274	0.048	0.00828	0.0556	0.00303	0.0242	0.0482	0.0196
**295**	0.0149	0.0269	0.0296	0	0.0629	0.00309	0.00471	0.0668	0.0121
**342**	0.0123	0.0231	0.0123	0	0.0696	0.0133	0	0.0804	0.0109
**396**	0.00789	0.0159	0.00236	0	0.0652	0.0253	0	0.0923	0.0126
**459**	0.00357	0.00818	0	0	0.0463	0.0278	0	0.0997	0.0148
**531**	9.17E-4	0.00272	0	0	0.0217	0.0173	0	0.0913	0.0163
**615**	7.72E-5	4.31E-4	0	0	0.0048	0.00466	0	0.0656	0.0152
**712**	0	0	0	0	0	0	0	0.0346	0.0102
**825**	0	0	0	0	0	0	0	0.0116	0.00406
**955**	0	0	0	0	0	0	0	0.00182	6.97E-4
**1110**	0	0	0	0	0	0	0	0	0
**1280**	0	0	0	0	0	0	0	0	0
**1480**	0	0	0	0	0	0	0	0	0
**1720**	0	0	0	0	0	0	0	0	0
**1990**	0	0	0	0	0	0	0	0	0
**2300**	0	0	0	0	0	0	0	0	0
**2670**	0	0	0	0	0	0	0	0	0
**3090**	0	0	0	0	0	0	0	0	0
**3580**	0	2.17E-4	0	0	0	0	0	0	0
**4150**	0	0.00224	0	0	0	0	0	0	0
**4800**	0	0.00933	0	0	0	0	0	0	0
**5560**	0	0.0128	0	0	0	0	0	0	0
**6440**	0	0.0055	0	0	0	0	0	0	0

**7460**	0	0	0	0	0	0	0	0	0
**8630**	0	0	0	0	0	0	0	0	0

	**0wks 33% RH**	**0wks7 4% RH**	**6wks 4℃ 33% RH**	**6wks 37℃ 33% RH**	**6wks 4℃ 74% RH**	**6wks 37℃ 74% RH**	**10wks 4℃ 33% RH**	**10wks 37℃ 33% RH**	**10wks 4℃ 74% RH**	**10wks 37℃ 74% RH**
**0**	0	0	0	0	0	0	0	0	0	0
**0**	0	0	0	0	0	0	0	0	0	0
**0**	0	0	0	0	0	0	0	0	0	0
**0**	0	0	0	0	0	0	0	0	0	0
**0**	0	0	0	0	0	0	0	0	0	0
**0**	0	0	0	0	0	0	0	0	0	0
**0**	0	0	0	0	0	0	0	0	0	0
**0**	0	0	0	0	0	0	0	0	0	0
**0**	0	0	0	0	0	0	0	0	0	0
**0**	0	0	0	0	0	0	0	0	0	0
**0**	0	0	0	0	0	0	0	0	0	0
**0**	0	0	0	0	0	0	0	0	0	0
**0**	0	0	0	0	0	0	0	0	0	0
**0**	0	0	0	0	0	0	0	0	0	0
**0**	0	0	0	0	0	0	0	0	0	0
**0**	0	0	0	0	0	0	0	0	0	0
**0**	0	0	0	0	0	0	0	0	0	0
**0**	0	0	0	0	0	0	0	0	0	0
**0**	0	0	0	0	0	0	0	0	0	0
**0.849**	0	0	0	0	0	0	0	0	0	0
**8.07**	0	0	0	0	0	0	0	0	0	0
**20.9**	3.77	0	0	0	0	0	0	0	0	0
**26.1**	15.9	6.99	0	0	0	0	0	0	0	0
**19.7**	28.1	22.7	0	0	0	0	0	0	0	0
**10.8**	27.3	30.3	0	0	0	0	0	0	0	0

**4.84**	16	22.7	0	0	0	0	0	0	0	0
**2.29**	5.58	11	0	0	0	0	0	0	0	0
**1.53**	0.892	3.31	0	0	0	0	0	0	0	0
**1.23**	0	0.472	0	0	0	0	0	0	0	0
**0.856**	0	0	0	0	0	0	0	0	0	0
**0.446**	0	0	2.79	0	0	0	0	0	0	0
**0.152**	0	0.00758	10.5	0	0	0	0	0	0	0
**0.0372**	0	0.063	17.3	4.53	0	0.958	0	0	0	0.958
**0.0644**	0	0.182	16.3	16.8	6.7	6.25	0.938	0	0	6.25
**0.151**	0.00948	0.291	12.2	25.1	23.3	15.2	8.68	0	0	15.2
**0.223**	0.0663	0.325	6.23	19.4	30.4	20	21.5	2.94	0	20
**0.247**	0.176	0.294	2.17	8.02	17.7	16.9	23.8	14.5	1.36	16.9
**0.222**	0.261	0.233	0.398	1.44	3.93	10.2	13.5	26	6.89	10.2
**0.168**	0.267	0.171	0.0956	0	0	4.45	3.78	21	13.9	4.45
**0.109**	0.211	0.121	0.313	0	0	1.26	0.326	7.48	14.9	1.26
**0.0595**	0.137	0.0859	0.492	0	0	0.174	0	0.876	9.47	0.174
**0.0258**	0.0722	0.0628	0.472	0	0	0	0	0	3.52	0
**0.00778**	0.0298	0.0483	0.313	0	0	0.0715	0	0	0.595	0.0715
**0.00218**	0.0084	0.0399	0.135	0.492	0	0.36	0	0	0	0.36
**0.00795**	0.00117	0.0365	0.0282	0.54	0.164	0.957	0	0	0	0.957
**0.0282**	0.00119	0.0387	0	0.78	1.99	1.76	0.259	0	0	1.76
**0.0637**	0.0104	0.0458	0.17	0.48	5.6	2.41	1.88	0	0	2.41
**0.107**	0.038	0.0536	0.906	0.72	6.43	2.58	5.36	0.458	0	2.58
**0.145**	0.0889	0.0591	2.46	0.35	3.2	2.23	8.26	3.19	1.15	2.23
**0.167**	0.157	0.0619	4.57	0.389	0.544	1.61	7.39	7.89	5.29	1.61
**0.16**	0.225	0.0617	6.44	0	0	1.12	3.63	9.22	11.1	1.12

**0.121**	0.269	0.0561	7.09	0	0	1.17	0.75	5.23	14.2	1.17
**0.0622**	0.228	0.0436	5.34	0	0	1.44	0	1.17	11.3	1.44
**0.016**	0.108	0.0252	2.11	0	0	1.28	0	0	5.22	1.28
**4.63E-4**	0.0262	0.0079	0.279	0	0	0.984	0	0	1.04	0.984
**0**	0.00575	6.36E-4	0.015	0	0	0.858	0	0	0	0.858
**0**	6.26E-4	0	0	0	0	0.732	0	0	0	0.732
**0**	0	0	0	0	0	0.542	0	0	0	0.542
**0**	0	0	0	0	0	0.297	0	0	0	0.297
**0**	0	0	0	0	0	0.0975	0	0	0	0.0975
**0**	0	0	0	0	0	0.0127	0	0	0	0.0127
**0**	0	0	0	0	0	0	0	0	0	0
**0**	0	0	0	0	0	0	0	0	0	0
**0**	0	0	0	0	0	0.0974	0	0	0	0.0974
**0**	0	0	0	0	0	1.11	0	0	0	1.11
**0**	0	0.00883	0	0	0	1.94	0	0	0	1.94
**0**	0	0.0177	0	0	0	0.92	0	0	0	0.92
**0**	0	0.00883	0	0	0	0	0	0	0	0
**0**	0	0	0	0	0	0	0	0	0	0

Table.4. Changes in PPI surface hydrophobicity

H_0	-20 °C			4 °C			37 °C			37 °C AP		
0 wks	601.25	581.56	591.33	601.25	581.56	591.33	601.25	581.56	591.33	601.25	581.56	591.33
2 wks	592.26	578.45	585.28	583.01	574.68	578.77	564.81	549.36	557.01	541.81	523.94	532.80
4 wks	569.61	558.68	564.07	533.87	531.16	532.44	520.27	493.76	506.94	484.61	481.74	483.10
6 wks	491.01	467.5	479.18	467.05	436.94	451.92	424.2	420.57	422.31	406.69	397.62	402.08
8 wks	397.57	390.82	394.12	390.29	380.00	385.07	378.48	369.87	374.1	377.26	361.21	369.16
10 wks	394.30	383.89	389.02	383.63	376.76	380.12	372.57	357.96	365.19	363.16	341.89	352.45

	4 °C 33%RH			37 °C 33%RH			4 °C 74%RH			37 °C 74%RH		
0 wks	545.65	525.52	535.51	545.65	525.52	535.51	530.87	515.66	523.19	530.87	515.66	523.19
2 wks	519.16	505.69	512.35	506.02	493.97	499.92	495.56	489.07	492.24	498.55	479.22	488.81
4 wks	483.89	466.56	475.15	472.35	468.44	470.32	421.13	410.6	415.79	372.3	360.07	366.11
6 wks	416.91	409.9	413.33	384.3	377.61	380.88	380.91	360.24	370.5	340.82	322.13	331.4

8 wks	381.59	378.4	379.92	366.56	354.61	360.51	338.31	331.48	334.82	314.74	306.43	310.51
10 wks	332.39	327.98	330.11	322.62	320.05	321.26	316.17	307.56	311.79	302.88	299.75	301.24

Table.5. Changes in PPI EAI

EAI (m^2/g)	-20 °C			4 °C			37 °C			37 °C AP		
0 wks	47.78	45.75	46.69	47.78	45.75	46.69	47.78	45.75	46.69	47.78	45.75	46.69
2 wks	47	44.57	45.71	45.66	44.29	44.9	39.74	38.76	39.19	37.06	36.76	36.85
4 wks	43.8	41.68	42.65	42.56	41.61	42.01	34.95	32.34	33.57	31.48	30.36	30.83
6 wks	36.57	35.59	35.99	36.15	32.13	34.05	30.97	28.47	29.63	26.62	25.35	25.91
8 wks	30.81	28.56	29.61	30.23	27.29	28.69	26.68	24.76	25.66	23.59	22.7	23.1
10 wks	28.14	25.98	26.97	27.28	24.88	25.99	23.55	20.97	22.18	20.62	20.38	20.41

	4 °C 33%RH			37 °C 33%RH			4 °C 74%RH			37 °C 74%RH		
0 wks	50.39	49.86	50.05	50.39	49.86	50.05	47.91	46.43	47.11	47.91	46.43	47.11
2 wks	48.39	46.83	47.52	47.69	45.23	46.42	45.58	44.22	44.81	40.98	37.87	39.35
4 wks	43.16	41.83	42.45	42.36	41.66	41.92	40.9	38.24	39.51	36.14	35.09	35.57
6 wks	37.61	35.83	36.66	35.1	34.23	34.59	34.85	33.31	34.02	33.25	32.33	32.73
8 wks	32.82	31.58	32.11	31.16	30.92	30.98	30.91	29.93	30.36	28.99	27.87	28.34
10 wks	31.46	28.85	30.08	29.32	28.85	29.01	27.56	26.23	26.82	24.3	22.59	23.4

Table.6. Changes in PPI ESI

**ESI (min)**	**-20 °C**			**4 °C**			**37 °C**			**37 °C AP**		
**0 wks**	25	24.64	24.76	25	24.64	24.76	25	24.64	24.76	25	24.64	24.76
**2 wks**	24.79	24.12	24.38	23.58	22.64	23.05	22.68	21.99	22.29	20.33	19.83	20.02
**4 wks**	24.22	23.2	23.62	22.67	21.63	22.09	20.42	18.9	19.57	17.88	17.03	17.41
**6 wks**	17.39	16.64	16.94	16.11	15.29	15.61	14.81	13.76	14.21	13.35	12.73	12.95
**8 wks**	16.18	15.56	15.81	15.59	14.88	15.16	14.05	13.39	13.63	12.33	11.95	12.07
**10 wks**	15.65	14.81	15.14	14.03	12.57	13.21	12.64	11.75	12.11	12.13	10.97	11.49

	**4 °C 33%RH**			**37 °C 33%RH**			**4 °C 74%RH**			**37 °C 74%RH**		
**0 wks**	20.28	19.95	20.04	20.28	19.95	20.04	20.04	19.78	19.82	20.04	19.78	19.82
**2 wks**	19.91	19.69	19.71	19.12	18.7	18.85	18.58	18.39	18.44	17.96	17.74	17.79
**4 wks**	18.07	17.85	17.9	16.54	16.35	16.37	15.89	15.6	15.67	15.25	15.00	15.05
**6 wks**	16.32	15.94	16.07	16.04	15.76	15.81	15.27	14.89	15.02	13.99	13.83	13.82
**8 wks**	15.13	14.84	14.91	13.3	13.09	13.15	12.26	11.94	12.05	11.23	10.91	11.01
**10wks**	13.43	13.2	13.27	11.65	11.45	11.49	11.11	10.71	10.84	10.31	9.96	10.09

Table.7. Changes in PPI secondary structures (α–helix and β–sheet)

	α–helix (%)			β–sheet (%)		
**0 wks**	14.79	14.71	14.78	36.73	36.71	36.84
**6wks -20℃**	14.64	14.51	14.59	36.49	36.41	36.33
**6 wks 4℃**	14.53	14.36	14.4	36.17	36.09	36.07
**6 wks 37℃**	14.31	14.42	14.44	35.99	35.95	35.91
**6 wks 37℃CAP**	14.29	14.23	14.2	35.65	35.63	35.58
**10wks-20℃**	14.41	14.31	14.33	35.69	35.58	35.53
**10wks -4℃**	14.39	14.28	14.26	35.58	35.48	35.47
**10wks 37℃**	14.32	14.28	14.27	35.35	35.3	35.28
**10wks37℃CAP**	14.26	14.12	14.16	35.2	35.14	35.17

	α–helix (%)			β–sheet (%)		
**0wks 33%RH**	14.78	14.62	14.67	36.7	36.57	36.53
**0wks 74%RH**	14.7	14.51	14.56	36.6	36.48	36.45
**6wks4℃33% RH**	14.55	14.42	14.47	36.12	35.99	35.98
**6wks37℃33%RH**	14.44	14.29	14.32	35.79	35.72	35.71
**6wks4℃74% RH**	14.35	14.28	14.27	35.56	35.4	35.45
**6wks37℃74%RH**	14.3	14.11	14.16	35.36	35.23	35.19
**10wks4℃33%**	14.39	14.3	14.3	35.4	35.32	35.33
**10wks37℃33%RH**	14.36	14.21	14.24	35.24	35.18	35.15
**10wks4℃74% RH**	14.24	14.13	14.11	35.02	34.93	34.93
**10wks37℃74%RH**	14.13	14.07	14.1	34.56	34.4	34.45

Table.8. Changes in PPI secondary structures (β–turn and Random coil)

	β–turn (%)			Random coil (%)		
**0 wks**	34.2	34.31	34.18	14.28	14.27	14.2
**6wks -20℃**	34.59	34.74	34.77	14.28	14.34	14.31
**6 wks 4℃**	34.91	35.04	35.08	14.39	14.51	14.45
**6 wks 37℃**	35.21	35.15	35.12	14.49	14.48	14.53

**6wks37℃AP**	35.39	35.44	35.46	14.67	14.7	14.76
**10wks-20℃**	35.28	35.41	35.42	14.62	14.7	14.72
**10wks -4℃**	35.3	35.45	35.48	14.73	14.79	14.79
**10wks 37℃**	35.49	35.62	35.63	14.84	14.8	14.82
**10wks37℃AP**	35.72	35.85	35.83	14.82	14.89	14.84

	**β–turn (%)**			**Random coil (%)**		
**0wks 33%RH**	34.31	34.45	34.47	14.21	14.36	14.33
**0wks 74%RH**	34.46	34.6	34.59	14.24	14.41	14.4
**6wks4℃ 33% RH**	35.02	35.15	35.22	14.31	14.44	14.33
**6wks37℃33%RH**	35.43	35.54	35.53	14.34	14.45	14.44
**6wks4℃ 74% RH**	35.67	35.8	35.75	14.42	14.52	14.53
**6wks37℃74%RH**	35.89	36.04	36.01	14.45	14.62	14.64
**10wks4℃ 3%RH**	35.76	35.85	35.88	14.45	14.53	14.49
**10wks37℃33%RH**	35.96	36.04	36.06	14.44	14.57	14.55
**10wks4℃ 74% RH**	36.23	36.39	36.37	14.51	14.55	14.59
**10wks37℃74%RH**	36.66	36.76	36.8	14.65	14.77	14.65
